# Honduran nurses’ perceptions among the quality of care for stroke patients: a qualitative study

**DOI:** 10.1590/1518-8345.7300.4419

**Published:** 2025-01-27

**Authors:** Sandra Patricia Nájera Figueroa, Oscar Fidel Antunez Martinez, Perla Simons Morales

**Affiliations:** 1 National Autonomous University of Honduras, School of Nursing, Tegucigalpa, Francisco Morazán, Honduras.; 2 National Taipei University of Nursing and Health Sciences, College of Nursing, Beitou District, Taipei, Taiwan

**Keywords:** Emergency Nursing, Cardiovascular Nursing, Stroke, Quality of Health Care, Honduras, Qualitative Interviews

## Abstract

**Objective:**

to explore the nurses’ perceptions among the quality of care to stroke patients in a public hospital in Northern Honduras.

**Method:**

a descriptive phenomenological study was carried out. The data collection was conducted by means of depth- interviews to 20 general nurses from the emergency and clinical medicine departments from the Atlántida General Hospital. Data analysis was by thematic technique.

**Results:**

the research found three key themes, and 10 subthemes, which illustrated the quality of stroke patients care in a general hospital from Honduras. The finding points up the theme “negative outcomes regarding the structure dimension of the patient care units”, which means the failure to improve or maintain the quality of healthcare. Likewise, “positive outcomes regarding the nursing process”, defined as pleasant and helpful among the care of stroke patients, such as ethical and humanized caring, and activities on promoting patient self-care with involvement of the relatives.

**Conclusion:**

the results indicate that Honduran nurses do not have qualified graduate training in critical care, which is a limitation that compromises the quality of care. Therefore, it is recommended to have clear organization structures and better resources managing, consequently, it may increase user satisfaction, as well as reducing hospital stays.

## Highlights

**(1)** Honduran emergency departments do not have competent nurses in critical care.

**(2)** Humanized care increases the patient satisfactions.

**(3)** Nursing staff is overloaded due to the lack of support from other departments.

**(4)** It is suggested a framework for nursing quality of care to stroke patients.

**(5)** A challenge on stroke patient care is the lack of nursing staff for each shift.

## Introduction

A cerebrovascular disease (CVD) or stroke is an acute compromise of the cerebral perfusion or vasculature^(1)^. Over the past several decades, the incidence of stroke and mortality is decreasing, however, stroke is the leading cause of adult disability worldwide^(1)^. Given their substantial contribution towards the escalating costs of health care, stroke also generate a high socio-economic burden in the general population which highlight the importance for nursing research in relation to management and quality of care^(2)^. The quality of care consists of carrying out each of the patient-oriented activities through the application of science and technology in a way that maximizes its health benefits without proportionally increasing its risks^(3-5)^.

Health services in Latin America are lack of budgets, which limit the quality of care and the availability of resources, impacting user satisfaction; consequently, the management models of these services differ according to the socioeconomic and cultural context where they are developed. Similarly, the personnel who provide these services face many constraints, particularly in developing countries, where the performance of personnel is measured by the provision of services to a greater number of patients to the detriment of the quality of care^(6-8)^.

In Honduras, health facilities where care to stroke patients is provided by technical diploma nursing staff with technical skills, likewise by general nurses with only undergraduate performing various roles, are limited at the time of the development of their activities due to the shortage of equipment, supplies and human resources as a consequence of the low budgets available in public hospitals^(8-9)^. It should be noted that the care of these patients is not protocolized, there are no care plans oriented to this pathology, and no publications with Honduran nursing scope, which prevents the provision of care in an equitable, logical, and systematic way to offer, as far as possible, a better quality of care for the management of patients with this disease. Significant gaps in the treatment and caring of CVDs remain, therefore, this phenomenon needs to be explored with a qualitative approach to know the experiences and perceptions of nursing in stroke patients’ care.

The assessment of healthcare quality should be grounded in a clear conceptual and operational definition of what quality of care entails for nurses^(10)^. Typically, the effectiveness of medical care, measured by recovery, functional restoration, and survival rates, is used as an indicator of the care’s quality^(10)^. Furthermore, Donabedian’s model proposed three approaches to assess the quality of care: structure, process, and outcome^(11)^. In this study, the emphasis was on structure and process, since the qualitative approach allows exploring the perceptions of the participants of these elements and does not seek to quantify the results. It is worth noting that Donabedian makes a judgment on a phrase: a good structure increases the possibility of a good process, and consequently, it increases the possibility of a good outcome^(12-14)^.

Thus, the overall purpose of this study was to explore the Honduran nurses’ perceptions among the quality of stroke patients care in a public hospital in Northern Honduras. It is expected with the finding of this study to contribute the policymaking and caring protocols development regarding the attention of nurses to stroke patients.

## Method

### Study design and scenario

This descriptive phenomenological study aimed to explore the perceptions of the nursing staff regarding the quality of care provided to CVD patients in the Emergency and Clinical Medicine departments at Atlántida General Hospital (HGA), La Ceiba, Honduras, followed by thematic analysis. This kind of approach offers a novel and innovative way to understand individual experiences and provides evidence for proposing evidence-based solutions, framework design, which may improve the quality of nursing care.

### Recruitment, selection criteria and sample definition

The participants were a total of 20 general nurses from the emergency and clinical medicine departments from the HGA, Atlántida, Honduras. The study used a purposeful sampling technique to recruit participants, by the emergency department’s nursing shift schedule, which was provided by the department director. According to Morse, it is recommended at least six and maximum 20 participants for phenomenological studies. Therefore, interviews occurred until data saturation was reached^(15)^, which means, a certain diversity of ideas has already been heard with each interview until other elements appeared.

The inclusion criteria were to be a nurse working different shifts and with at least one year of experience in the care of patients diagnosed with CVD in the emergency or clinical medicine HGA’s departments, and not be on vacation or incapacitated during data collection.

### Data collection procedure and instrument

Data was collected between August and October of 2022 in the native language of the participants (Spanish) through in-depth interviews. The interview guide contained five open-ended questions with the main study’s concepts, with an approximate duration of 20 to 45 minutes in a quiet and private space around the emergency department’s waiting room, since it allows to the participants to express their opinions and emotions comfortably. In addition, the instrument was validated by a committee expert in qualitative research methodology and with CVD or emergency nursing as research field.

The study’s main author, a nurse who did not work in the emergency department, carried out the data collection. However, she is a nurse from another department in the same hospital, then she is familiar to the personnel. The interviewer has experience developing semi-structured interviews, and she was guided by an expert in qualitative research during data collection. Likewise, the study was carried out in the HGA’s emergency department, due to it is a reference health institution for its level of complexity in providing free specialized health care to the most serious cases from northern Honduras.

### Data treatment and analysis

It was developed following Colaizzi’s seven steps, which consist of familiarization, identification of the main narratives, formulating meanings, grouping themes, developing an exhaustive description, producing the fundamental structure, and seeking structure’s verification^(16)^. Firstly, the data obtained will be transcribed, then, organized and coding by themes and sub-themes of logically analysis.

For the interpretation and implications of the theoretical framework, Streubert’s triangulation steps were followed^(17)^, which consist on reflect on the combined findings to draw conclusions about the quality of care for stroke patients and its impact on participants’ experiences and outcomes; identifying opportunities for quality improvement based on the insights gained from both phenomenological research and the Donabedian framework; considering the implications of the findings for healthcare policy, practice, education, and research. By following Donabedian’s model for quality of care^(11)^, Antunez Martinez’s nursing care model^(18)^, and this study findings, it was planned to design a theoretical framework for nursing care to stroke patients.

### Trustworthiness

The research project applied the following criteria for trustworthiness, which consists on credibility, appropriateness, auditability, and confirmability^(19-20)^. In order to comply with these elements, the following strategies were applied and are described below. For credibility, the negative cases’ strategy was used, which consists of comparing the different narratives with other elements under discussion in the search for data that are properly substantiated, that appear contradictory or that explain the emerging data.

Likewise, it was applied the strategy of member checking as a confirmability strategy, which consists in that the interpretations, themes, and conclusions are verified by the members where the data were originally obtained. For auditability, the audit trail strategy is used, which consists of keeping the recordings and notes related to the data collection for the next five years, and it is worth mentioning that the research process has counted on different expert committees that have followed up on the study’s development. For the suitability, the flexibility criteria are followed, which counts with the participation of different researchers with experience in the development of qualitative studies from different approaches, thus allowing the reasonable presentation of the data after discussions and consensus with those involved.

### Ethical aspects

The project was approved by the biomedical research ethics committee of the Faculty of Medical Sciences, Universidad Nacional Autonóma de Honduras (IRB 00003070; Approval code 027-2022), and authorization was granted to the HGA’s Department of Nursing. The latter gave written authorization to carry out the data collection, then, the participants signed the informed consent after the interviewer explained to them the aim of the study, and that their participation in the study would not put their integrity at risk. Therefore, the identities of the participants were coded as a protective method, using the letter P (participant) followed by the number of the interview.

## Results

A total of 20 general nurses participated as key informant during data collection. Their ages ranged between 24 to 55 years old; 10 were single, 5 married, 2 widowers, and 3 registered partnerships; 4 were males, and 16 females. Furthermore, regarding the educational level, all the participants were registered nurses with no graduate or specialization diploma. Furthermore, their work experience period was between 1 to 19 years in the selected departments.

We discussed our findings under 3 key themes, 10 subthemes, and 21 concepts that were brought up most by the participants reflecting about their perceptions regarding the quality of care provided by nursing personnel to patients with a CVD diagnosis in the Emergency and Clinical Medicine departments of the HGA ^(Figure 1)^. Following are available the thematic groups with their respective interpretations, and quotations. Themes and subthemes definitions are available in Figure 2.

**Figure 1:** - Summary of thematic groups of Honduran nurses’ perceptions among the quality of care for patients with cerebrovascular accident

**Themes**	**Sub-themes**	**Concepts**
1. Negative outcomes regarding the structure dimension of the patient care units	1.1 Unsanitary spaces	1.1.1 Ambiguous concurrent disinfection 1.1.2 Sporadic cleaning 1.1.3 Insufficient cleaning personnel
	1.2 Inadequate ventilation system	1.2.1 Failed air conditioners 1.2.2 Lack of windows 1.2.3 Noise pollution
	1.3 Limited dimensional space	1.3.1 Overcrowding 1.3.2 Vulnerable privacy
2. Challenges related to nursing care processes	2.1 Maintaining patient privacy during the performance of nursing interventions	2.1.1 Non-cooperation by relatives 2.1.2 Lack of portable partition
	2.2 Lack of supplies and equipment	
	2.3 Lack of specialized nursing personnel	2.3.1 Lack of professional autonomy 2.3.2 Basic care competencies
	2.4 Insufficient nursing personnel in rotating shifts	2.4.1 Shifts covered by nursing assistant students 2.4.2 Overloaded work
	2.5 Ambiguous support from other hospital departments	2.5.1 Delayed diagnoses 2.5.2 Prolonged treatments 2.5.3 Extended patient stays
3. Positive outcomes regarding the nursing process	3.1 Ethical and humanized Honduran nursing care	3.1.1 Empathy 3.1.2 Emotional support 3.1.3 Professional vocation 3.1.4 Labor commitment
	3.2 Discharge planning with incorporation of self-care	

**Figure 2:** - Definitions of the thematic groups regarding Honduran nurses’ perceptions among the quality of care for stroke patients

TG*	**D** ^†^
1. Negative outcomes regarding the structure dimension of the patient care units	Failure to improve or maintain the quality of nursing care to Honduran patients with CVD ^‡^ regarding the structure of the area where the patients are hospitalized.
1.1 Unsanitary spaces	Intra-hospital spaces without compliance of terminal and concurrent disinfection protocols by trained personnel.
1.2 Inadequate ventilation system	Patient units with scant freshening systems due to devices in poor condition or noisy, and without fresh air inlets.
1.3 Limited dimensional space	Inconvenient size of the units among the number of inpatients.
2. Challenges related to nursing care processes	Dispute the validity of nursing caring processes with high level of quality to patient with CVD ^‡^ in the public health system of middle-low-country.
2.1 Maintaining patient privacy during the performance of nursing interventions	Difficulties in maintaining patient security and confidentiality during complex care processes due to overcrowding and lack of protection supplies.
2.2 Lack of supplies and equipment	Not enough or it does not exist at all some supplies and equipment require for the caring processes of stroke patients, which means a risk in the patient successful recovery.
2.3 Lack of specialized nursing personnel	Inadequately trained nurses on stroke patient care, therefore, poor job experiences and possible increased levels of work-related stress, and low patient safety level.
2.4 Insufficient nursing personnel in rotating shifts	Not enough staff are hired for critical areas, and the workload intensity is too complex to be managed safely within the different shifts.
2.5 Ambiguous support from other hospital departments	A state of uncertainty intentionally, behavior pattern or situation that might be interpreted in a wrong way due to resistance to fulfilling their responsibilities from laboratory, images, and radiation departments. Consequently, it affects the patient diagnosis and caring plans, increasing the length of stay.
3. Positive outcomes regarding the nursing process	Experiences pleasant and helpful to nursing processes among the care of patient with CVD ^‡^ .
3.1 Ethical and humanized Honduran nursing care	Considering patient and family members’ health condition and feelings to adjust their behavior and caring to demonstrate healthcare system as reasonable and flexible. Therefore, Honduran nursing care is not supported on the institutional intentions, but on attitudes focused on the patient wellbeing.
3.2 Discharge planning with incorporation of self-care	Formal release of a patient from the hospital once they appear to be in better health, and suitable for outpatient from home with integration of the family in the care plans. Activities to promote self-care and prepare relatives are carried out throughout the entire hospitalization period and are the responsibility of the nursing staff.

*TG = Thematic Groups; ^†^D = Definitions; ^‡^CVD = Cerebrovascular Disease

### Negative outcomes regarding the structure dimension of the patient care units

Unsanitary spaces: The participants report that the services are inadequate since they lack a proper and frequent disinfection process. In other words, they do not comply with the number of concurrent and terminal disinfections required by the complexity of the emergency and clinical medicine departments. This situation is since the cleaning personnel is not assigned to stay in the departments, likewise, they perform concurrent disinfection from Monday to Friday, once per day. Meanwhile, on the other days, the patient’s family members are the ones who carry out these activities. This compromises the quality of the facilities because the patient’s relatives do not have the technical training to perform the proper disinfection activities. It should be noted that the frequency of terminal disinfections in the facility is not documented.

*There is a lack of hygiene... the cleaning staff cleans, but not thoroughly. Since we are in an emergency as soon as a patient with a cerebrovascular event arrives, other renal patients arrive and stain the walls, the beds, and not only that, but blood also remains on the floor.* (P1)

*It’s not right. Why they don’t come by cleaning this daily or every now and then, but when they have time... there should be one staff specifically for the emergency area and I haven’t seen one that’s here.* (P3)

*The cleaning personnel only in the morning from Monday to Friday... the same family members help us with disinfection.* (P14)

*The cleaning personnel as it is only one and they have more areas to clean.* (P17)

Inadequate ventilation system: The services do not have an adequate ventilation system, because the personnel report that they cannot open the only window available due to the risk of access to infectious vectors outside the hospital. Ventilation systems such as fans and air conditioners are not in good condition, so patients’ relatives need to bring their own supplies to improve the conditions of the patients’ stay. It should be noted, they do not use the ventilators that are in the services because this implies a risk of noise pollution and disharmonization of the environment related to the noise produced when the ventilators are in operation.

*The structure is deplorable because we do not have good ventilation. The air conditioners do not work... it gets ridiculously hot. This affects the personnel’s performance... there are windows, but they do not open because, as we are attached to another side, insects can get in, or when it is raining, that same wind brings in water.* (P1)

*The ward is not well conditioned... and that affects the patients because some of them suffocate, they are hot, even for us, the nurses... the area is not ventilated.* (P7)

*Each patient brings their fan ... but it is not enough, and our fan makes a lot of noise too. Using it is noise pollution.* (P13)

Limited dimensional space: The participants reported that the dimensions of the cubicles, as well as their number, are limited; this is because they are not commensurate with the number of patients attending the service. Thus, when the admission of patients is high, the nursing personnel is forced to place several patients in the same cubicle, not respecting the adequate distance between them. This situation is alarming because of the increased risk of nosocomial infections.

*The cubicles’ dimensions and the number of units available are not adequate... not only do we receive patients with stroke, but also with suspected COVID-19, which is the most frequent disease nowadays, and we do not have adequate distancing to prevent hospital-acquired infections.* (P1)

*More patients than necessary are placed in each cubicle, and this is due to the high admission of patients... The cubicles are too small in relation to the number of patients admitted. There is no space... sometimes because of the cubicles’ conditions, instead of helping the patient, the patient becomes more complicated.* (P8)

### Challenges related to nursing care processes

Maintaining patient privacy during the performance of nursing interventions: It is one of the challenges faced by the nursing staff in the care of stroke patients in the emergency and clinical medicine departments, the conditions of the services are not favorable for the high patients’ demand, so they are forced to place several patients in the same cubicle in different units remarkably close to each other. Then, family members and other visitors insist on staying in the unit and the nursing staff does not have screens to take care of the patients’ privacy, and this interferes with the quality of nursing care processes.

*In some cases, we have to do procedures in private to take care of the patient’s integrity, but the relatives do not want to leave the cubicle… in the other unit in the same cubicle there are other patients with their relatives, and we have to manage to take care of the patient’s privacy, although it is not always possible. (P4)*

*The problem is with the family members, even though we tell them that this is a restricted area there are always visitors and relatives with the patient. (P5)*

*There are no partitions at the moment, and there are quite a few patients and family members in the same cubicle. (P9)*

Lack of supplies and equipment: The departments lack medical-surgical equipment and supplies to provide quality nursing care to stroke patients. The hospital supplies some basic necessities; however, the quantity is not congruent with the number of patients attending the departments. Similarly, in relation to medical-surgical equipment, the departments do not have sufficient quantity, therefore, the nursing personnel are forced to travel to other services to request their borrowed equipment. This implies an expense of time and energy, not to mention that the other departments are temporarily short of the equipment necessary for the care of critical patients.

*We lack supplies. Many times, we do not even have basic things like syringes, intravenous catheters, intravenous solutions... The hospital administration replenishes the materials, but not enough... and we do not have the whole medical-surgical equipment that stroke patients need... we must request other departments to lend us their equipment. We lose time in those displacements.* (P1)

*We should have more medical-surgical equipment and supplies to provide better care to stroke patients, for me it is a challenge to work in these conditions... some supplies are lacking to provide good care.* (P3)

*What we do is to give the prescription to the family members so they may buy what they require.* (P12)

Lack of specialized nursing personnel: The clinical medicine and emergency departments do not have nursing personnel specialized in critical and emergency care, their competencies are limited to basic knowledge of care and most of the staff lack clinical experience. Consequently, the lack of specialized personnel causes the department’s nurses to depend on other disciplines for making decisions related to the care of stroke patients, losing their professional autonomy and leadership, which compromises the quality of care in the process dimension.

*The specialist physician is responsible for several complex procedures because we do not have specialist nurses, and that is a problem... because I, for example, am not going to be so attentive to the mechanical ventilator because, although I have basic knowledge, there should always be a specialist nurse taking care of patients with stroke, and we do not have one in the department.* (P4)

*The nursing personnel in the department are not specialists and lack experience, so they do not understand the complexity of a stroke patient. The quality of care is compromised, the tasks are simply being performed.* (P5)

*These patients with stroke need specialized care... We only have general nurses and not all of them are professionally trained because they are new personnel... there are patients who require care that only specialist nurses can perform. Then we become dependent on physicians for these processes. Nursing loses autonomy as a professional.* (P7)

Insufficient nursing personnel in rotating shifts: A notable challenge in the emergency and clinical medicine departments is the lack of nursing staff for each shift. Participants report that there are shifts where there is only one general nurse responsible for 14 critically ill patients, and at times she must share her responsibilities with students and nursing assistants because she alone cannot meet all the needs that patients present. Nevertheless, there are still things pending for the next shifts that face the same problem, and the quality of care for CVD patients is compromised.

*The students from the school of nursing assistants are collaborating by taking some shifts with us.* (P2)

*There is a lack of personnel. Sometimes there is only one nurse assistant, one nursing student, and a general nurse... when there are staff on vacation or on disability, there is only one person left per shift.* (P6)

*In the afternoon shifts to rotate the patient, it is quite difficult. Sometimes I am alone as a nurse, maybe I manage to get support from the family member, but still, it is complicated.* (P13)

*Sometimes there are 13 patients for one nurse, and I am talking about a department that takes care of patients in critical conditions. So, you can’t cope with so many patients ... there are many things pending.* (P14)

*We need more personnel, as we not only have stroke patients admitted for observation, but we also handle other patients who come in for other emergencies.* (P19)

Ambiguous support from other hospital departments: Participants describe difficulties in the process of diagnosis and treatment of CVD patients due to the ambiguous support of the supportive departments. Imaging staff do not perform all the necessary diagnostic procedures, in addition to having restricted service schedules. Similarly, laboratory personnel do not demonstrate availability to deliver requested test results in a timely manner. Therefore, the nursing personnel in the emergency and clinical medicine departments have to invest time and effort to facilitate the processes of the support departments to obtain the requested services.

*Sometimes it takes quite a long time to detect what type of CVD the patient has... Then we get behind... the X-Ray is not performed every day, they have a schedule, but sometimes they come and say they will be available until 6:00pm; Sometimes they come and say: today we are not doing X-Ray or CT.* (P5)

*The laboratory department fails us 100%, and we depend on them... They take a long time to deliver the results even though we help them to collect the samples [...] the pharmacy department only gives us the medicines for daily use according to the prescription, but if we have an emergency, we depend on whether the personnel want to give it or not. We waste a lot of time on that.* (P7)

### Positive outcomes regarding the nursing process

Ethical and humanized Honduran nursing care: Within the positive findings of the study in the process dimension is the humanized treatment with professional ethics by the nursing personnel to the CVD patient in the emergency and clinical medicine services. This treatment is based on empathy, considering the patients’ feelings. Transmitting positive energy to the patient by behaving in an encouraging manner, giving emotional support, and maintaining open channels of communication with both patients and family members. This type of behavior is present in nursing personnel with commitment with the patient recovery and vocation for their profession.

*Empathy... If the patient is conscious and looks at us with bad expressions, they would feel bad, so we always try to be cheerful and share positive energy to the patient.* (P1)

*I am a person who treats family members kindly... I try to treat the patient the way I would like my family to be treated... I would like to be seen the way I look at patients.* (P4)

*Stroke patients are vulnerable, so we have to pay them attention and give them as much support as possible, talk to them... give them encouragement,... act with empathy... give them emotional support... they must feel that empathy on the part of the nurses.* (P12)

*I feel good because I chose this profession that I like. So, I take care of stroke patients with the best attitude.* (P14)

Discharge planning with incorporation of self-care: The process of planning the patient’s discharge from the hospitalization service for the safe and autonomous return of the patient to his or her home, with the incorporation of the family in the care activities. In Honduras, nursing is responsible for educational and administrative activities related to the patient’s discharge.

*The patient and family members are taught the care that stroke patients need... we take our time so that they know what to do when they return home.* (P6)

**Figure 3:**
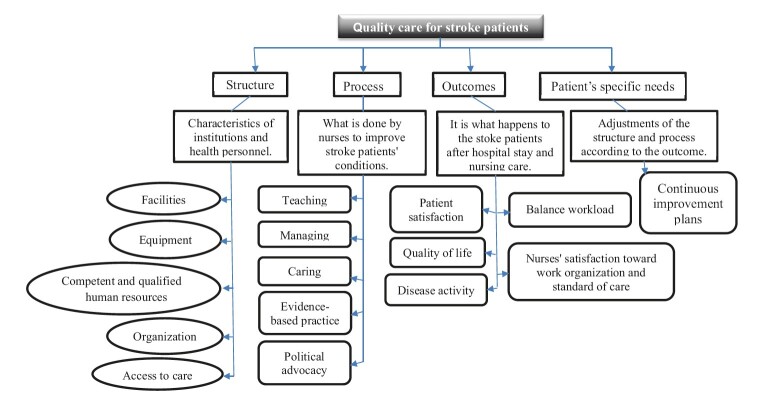
- Theoretical framework for nursing quality of care to stroke patients

Note: Authors’ own, based on study findings, Donabedian quality of care model, and Antunez Martinez nursing care model

By merging phenomenological research with Donabedian’s quality of care theory for stroke patients, we gained a thorough comprehension of both patient experiences and factors impacting healthcare quality. Through triangulation, we pinpointed patterns, themes, and inconsistencies in the data, informing suggestions for enhancing stroke patient care quality. Comparing and blending findings from phenomenological research, the Donabedian framework and Antunez Martinez’s nursing care model enabled us to grasp the quality of stroke patient care from both participant perspectives and a quality improvement standpoint ^(Figure 3)^.

## Discussion

In this study, three themes were obtained: *Negative outcomes regarding the structure dimension of the patient care units*, *Challenges related to nursing care processes*, and *Positive outcomes regarding the nursing process*. This study with the sample of Honduran general nurses from emergency and clinical medicine wards, supports the results of studies from high income countries exploring the experiences of health personnel among the quality of care to stroke patients.

Stroke patients require specialized care, as well as the necessary equipment for an expedited, effective, and timely diagnosis. However, Honduran emergency and clinical medicine departments do not have all the equipment needed to provide the care required for stroke patients, causing a delayed response in the diagnosis of this type of patient, depending on the type (hemorrhagic or ischemic). Consequently, the nursing care process must proceed further. These shortcomings have a direct effect on the quality of care for patients with this diagnosis, both because of the lack of equipment and competent nursing personnel in charge of caring for them.

The above finding is congruent with a study conducted in the north of England, where the results highlight the lack of resources and environment as a barrier to quality care and implementation of intervention guidelines^(21)^. It should be noted that most of the studies regarding quality of care are from developed countries, thus, their focus is on the personnel organization and their specific competences and not the lack of supplies and inadequate inpatient spaces^(22-23)^. Except for a study conducted in Sweden, which highlights environmental issues, the research does not address the specific care needs of stroke patients^(24)^. It is recommended to continue exploring this issue in low-middle incomes countries’ health systems.

In relation to the process dimension, the nursing care that the participants reported to be developed in the service were those of integrated care and general nursing competencies. Therefore, some complex procedures are performed by the medical staff, which implies a loss of space for the nursing personnel as autonomous professional and leader of the care processes.

In relation to the care of stroke patients^(25)^ mentioned, it is necessary strong clinical skills and competences in interaction with the patient and their relatives. Therefore, continuing training is relevant for developing healthcare professionals’ competence in specialized nursing care as part of the appropriate quality of care^(26-28)^.

A cross-sectional study developed in England, Belgium, Finland, Spain, Ireland, Iceland, and Switzerland, point out the reduction of complex clinical responsibilities on nursing personnel as well as the overburdening of physician staff may contribute to preventable deaths, eroding the quality and safety of hospital care^(29)^. It is necessary to apply studies to explore the dynamics of nursing work organization with other health professionals in order to understand this phenomenon, as well as to apply quantitative studies to measure and describe nurses’ competencies in Latin-American public health system. Subsequently, it is recommended the design of public policies that guarantee more spaces for advanced interventions for nursing personnel, as well as the promotion of specialized competencies in the critical care of stroke patients.

Similarly, ambiguous support from other services such as laboratory and X-ray services is highlighted among the participants’ narratives. This fact is congruent with the results of other studies that highlight as a barrier to quality care the delayed response of support services for stroke’s diagnosis and treatment^(30-32)^. This issue, as well as our study, is due to the resistance of health personnel to change their attitude and the limited number of personnel.

The above phenomenon is not congruent with principles of Donabedian’s model^(11)^, who states that the evaluation of the quality of the process is the set of actions that must be carried out to reach a specific result. It is a complex process of interaction or communication between the patient and the healthcare personnel, in addition to the technology used, the specialized competencies required by the patient by the health personnel, and the timely execution of the procedures for diagnosis and treatment of the patients should play a relevant role.

Among the positive findings, the professional ethical posture and humanized treatment of stroke patients by the nursing staff stand out. Likewise, stroke patients need better access to emotional support by health personnel, including information and advice, both patients and family members, which is congruent to Honduran nurses’ actions focused on humanized treatment and strengthening self-care during the discharge process^(32-34)^. More research is required to establish the effectiveness of nurses’ emotional support, and their competences mental health.

The importance of patient and family education during discharge is reinforced by an author who mentions that the recovery process after a stroke is complex and tedious, therefore, a good orientation on the next steps to follow is needed^(35)^. This educational activity is prioritized by the participants, subsequently, it is recommended to explore those activities and established protocol of stroke patients’ discharge following nursing perspectives.

The limitations included the fact that nursing personnel working in HGA’s emergency and clinical medicine departments were not accustomed to participating in this type of research approach; Consequently, it caused a certain degree of stress because it was completely recorded, limiting at times the spontaneous and complete expression of the answers to the posed questions.

Similarly, the participant’s preparation level was a limitation. It is considered that developing this methodology with critical care and emergency nurses may contribute to the design of an expanded model for nursing practice in the care of stroke patients. Finally, the study was carried out in a single location, which limits a more general overview of the phenomenon in Honduras.

Within the strengths was the number of participants, which allowed for increased representativeness of the study. Likewise, the person responsible for conducting the interviews was a nurse with experience in qualitative studies who works in the same institution, which facilitated the acceptance of the people contacted to participate in the study.

## Conclusion

Honduran nurses’ experiences revealed a challenging healthcare landscape for stroke patients. The existing infrastructure falls short of meeting basic quality standards, jeopardizing patient safety. This sheds light on the realities of hospital services in Honduras and the demanding working conditions faced by nurses, often overlooked but offering insights applicable to improving situations globally. Nurses in Honduran emergency and clinical departments lack specialized training in critical care, posing a limitation that impacts the quality of care provided, given the complexity of stroke patient needs. There’s a pressing need to ensure emergency departments are staffed with individuals equipped to lead care processes effectively. Despite these challenges, a positive note emerges in the dimension of humanization and ethical treatment, with the nursing team demonstrating crucial support and empathy, factors that warrant consideration.

By triangulating phenomenological research with Donabedian’s theory, it is possible to gain a deeper understanding of the complex interplay between nurses’ experiences and healthcare quality in the context of stroke care, ultimately leading to more informed and effective interventions to improve patient outcomes and satisfaction. Policy makers and health managers should recognize the need of incorporating nurses with advanced competencies in the care process in order to guarantee the recovery of the user with highest level of quality of nursing care in middle-low-income countries. Similarly, have clear organization structures and better resources managing. These types of strategies will bring greater benefits to public health institutions, and will increase user satisfaction, as well as reducing hospital stays.
