# Supplementary material for: Honduran nurses’ perceptions among the quality of care for stroke patients: a qualitative study
Source: Rev Lat Am Enfermagem. 2025 Jan 27;33:e4419. doi: 10.1590/1518-8345.7300.4419 (PMC11774144; doi:10.1590/1518-8345.7300.4419)
Supplement: Translation

# Translation: Percepciones de enfermeros hondureños sobre la calidad de la atención al paciente con accidente cerebrovascular: estudio cualitativo

**DOI:** 10.1590/1518-8345.7300.4418

**Authors:** Sandra Patricia Nájera Figueroa, Oscar Fidel Antunez Martinez, Perla Simons Morales

**Affiliations:** 1 National Autonomous University of Honduras, School of Nursing, Tegucigalpa, Francisco Morazán, Honduras.; 2 National Taipei University of Nursing and Health Sciences, College of Nursing, Beitou District, Taipei, Taiwan

**Keywords:** Enfermería de Urgencia, Enfermería Cardiovascular, Accidente Cerebrovascular, Calidad de la Atención en Salud, Honduras, Investigación Cualitativa

## Abstract

**Objetivo:**

explorar las percepciones de los enfermeros sobre la calidad de la atención a pacientes con accidente cerebrovascular en un hospital público del norte de Honduras.

**Método:**

se realizó un estudio fenomenológico descriptivo. La recolección de datos se realizó a través de entrevistas en profundidad a 20 enfermeros generales de los servicios de emergencia y clínica médica del Hospital General de Atlántida. El análisis de los datos se realizó mediante la técnica temática.

**Resultados:**

a partir de la investigación surgieron tres temas principales y 10 subtemas que ilustraron la calidad de la atención a los pacientes con ACV en un hospital general de Honduras. El hallazgo apunta al tema “resultados negativos con respecto al tamaño de la estructura de las unidades de atención al paciente”, lo que significa que hay problemas para mejorar o mantener la calidad de la atención médica. Por otro lado, se obtuvieron “resultados positivos con respecto al proceso de enfermería”, definido como placentero y útil para el cuidado del paciente con ACV, como cuidados éticos y humanizados y actividades para promover el autocuidado del paciente con la participación de los familiares.

**Conclusión:**

los resultados indican que los enfermeros hondureños no cuentan con formación de posgrado calificada en cuidados críticos, lo cual es una limitación que compromete la calidad de la atención. Por lo tanto, se recomienda contar con estructuras organizativas claras y una mejor gestión de los recursos, lo que, en definitiva, puede aumentar la satisfacción de los usuarios y reducir el tiempo de estancia hospitalaria.

## Destacados

**(1)** Los servicios de emergencias en Honduras no cuentan con enfermeros competentes en cuidados críticos.

**(2)** La atención humanizada aumenta la satisfacción del paciente.

**(3)** El equipo de enfermería está sobrecargado por la falta de ayuda de otros departamentos.

**(4)** Se sugiere contar con una estructura que permita brindar atención de enfermería de calidad a los pacientes con ACV.

**(5)** La falta de personal de enfermería para cada turno es un desafío para la atención a pacientes con ACV.

## Introducción

La enfermedad cerebrovascular (ECV) o accidente cerebrovascular (ACV) es un deterioro agudo de la perfusión o circulación cerebral^(1)^. En las últimas décadas, la incidencia y la mortalidad por accidentes cerebrovasculares han ido disminuyendo; sin embargo, el ACV es la principal causa de discapacidad en adultos en todo el mundo^(1)^. Dada su contribución sustancial al aumento de los costos de la atención médica, el accidente cerebrovascular también genera una alta carga socioeconómica en la población general, lo que destaca la importancia de la investigación de enfermería sobre la gestión y la calidad de la atención^(2)^. La calidad de la atención consiste en realizar cada una de las actividades dirigidas al paciente mediante la aplicación de la ciencia y la tecnología de forma que maximice los beneficios para la salud sin aumentar proporcionalmente los riesgos^(3-5)^.

Los servicios de salud en América Latina sufren de falta de presupuesto, lo que limita la calidad de la atención y la disponibilidad de recursos, y afecta la satisfacción de los usuarios; por ello, los modelos de gestión de dichos servicios difieren según el contexto socioeconómico y cultural en el que se implementan. Además, el personal que brinda esos servicios enfrenta muchas restricciones, especialmente en los países en desarrollo, donde el desempeño del personal se mide en función de la prestación de servicios a un mayor número de pacientes, en detrimento de la calidad de la atención^(6-8)^.

En Honduras, los establecimientos de salud donde la atención a pacientes con ACV la brinda el personal de enfermería con título técnico y habilidades técnicas, y los enfermeros generales que tienen solo el título de grado que desempeñan diversas funciones, tienen limitaciones a la hora de llevar a cabo sus actividades debido a la escasez de equipamiento, insumos y recursos humanos como consecuencia del bajo presupuesto disponible en los hospitales públicos^(8-9)^. Cabe señalar que no hay protocolos para brindarle atención a estos pacientes, no hay planes de atención orientados a esta patología y no hay publicaciones dedicadas a la enfermería hondureña, lo que impide brindar atención de manera equitativa, lógica y sistemática para contar, dentro de lo posible, con una mejor calidad asistencial para el manejo de los pacientes con esta enfermedad. Siguen existiendo lagunas importantes en el tratamiento y la atención de las ACV, por lo que es necesario explorar este fenómeno con un enfoque cualitativo para comprender las experiencias y percepciones de los enfermeros en la atención de los pacientes con ACV.

La evaluación de la calidad de la atención médica debe basarse en una definición conceptual y operativa clara de lo que significa para el enfermero calidad de la atención^(10)^. Normalmente, la eficacia de la atención médica, medida por las tasas de recuperación, restauración funcional y supervivencia, se utiliza como indicador de la calidad de la atención^(10)^. Además, el modelo de Donabedian propuso tres enfoques para evaluar la calidad de la atención: estructura, proceso y resultado^(11)^. En este estudio se puso énfasis en la estructura y el proceso, dado que el enfoque cualitativo permite explorar las percepciones de los participantes sobre esos elementos y no busca cuantificar los resultados. Cabe mencionar que Donabedian afirmó que: una buena estructura aumenta la posibilidad de un buen proceso y, por ende, aumenta la posibilidad de un buen resultado^(12-14)^.

Por lo tanto, el objetivo general de este estudio fue explorar las percepciones de enfermeros hondureños sobre la calidad de la atención a pacientes con accidente cerebrovascular en un hospital público del norte de Honduras. Se espera que los resultados de este estudio contribuyan a formular políticas y desarrollar protocolos de atención para la atención de enfermería a pacientes con ACV.

## Método

### Diseño y escenario del estudio

Este estudio fenomenológico descriptivo tuvo como objetivo explorar las percepciones del equipo de enfermería sobre la calidad de la atención brindada a pacientes con ECV en los departamentos de emergencias y clínica médica del Hospital General de Atlántida (HGA), La Ceiba, Honduras, seguido de un análisis temático. Este tipo de enfoque ofrece una forma nueva e innovadora de comprender las experiencias individuales y proporciona evidencia para proponer soluciones basadas en evidencia, diseño de estructura, que pueden mejorar la calidad de la atención de enfermería.

### Reclutamiento, criterios de selección y definición de la muestra

Los participantes eran en total 20 enfermeros generales de los departamentos de emergencias y clínica médica del HGA, Atlántida, Honduras. El estudio utilizó una técnica de muestreo intencional para reclutar participantes en los horarios de los turnos de enfermería del departamento de emergencias, proporcionado por el director del departamento. Según Morse, para los estudios fenomenológicos se recomienda un mínimo de seis y un máximo de 20 participantes. Por lo tanto, las entrevistas se realizaron hasta alcanzar la saturación de datos^(15)^, lo que significa que en cada entrevista ya se escuchaba cierta diversidad de ideas hasta que aparecían otros elementos.

Los criterios de inclusión fueron: ser enfermero que trabajaba en diferentes turnos y con al menos un año de experiencia en la atención a pacientes diagnosticados con ECV en los servicios de emergencia o clínica médica del HGA, y no estar de vacaciones o incapacitado durante la recolección de datos.

### Procedimiento e instrumento de recolección de datos

Los datos se recolectaron entre agosto y octubre de 2022 en el idioma nativo de los participantes (español) mediante entrevistas en profundidad. La guía de la entrevista contenía cinco preguntas abiertas con los conceptos principales del estudio, la entrevista tuvo una duración aproximada de 20 a 45 minutos en un espacio tranquilo y privado cercano a la sala de espera del departamento de emergencias, dado que eso les permite a los participantes expresar sus opiniones y emociones cómodamente. Además, el instrumento fue validado por un comité especializado en metodología de investigación cualitativa y cuyo campo de investigación es la enfermería de emergencia o de ECV.

La autora principal del estudio, una enfermera que no trabajaba en el departamento de emergencias, realizó la recolección de datos. Sin embargo, ella es enfermera en otro departamento del mismo hospital y, por lo tanto, está familiarizada con el personal. La entrevistadora tiene experiencia en el desarrollo de entrevistas semiestructuradas y recibió orientación de un especialista en investigación cualitativa durante la recolección de datos. Además, el estudio se realizó en el servicio de emergencias del HGA, debido a que es una institución de salud de referencia por su nivel de complejidad para brindar atención médica especializada gratuita a los casos más graves del norte de Honduras.

### Procesamiento y análisis de datos

Fue desarrollado siguiendo los siete pasos de Colaizzi, que consisten en familiarizarse, identificar las principales narrativas, formular significados, agrupar los temas, desarrollar una descripción exhaustiva, elaborar la estructura fundamental y verificar la estructura^(16)^. En primer lugar, se transcribirán los datos obtenidos para, posteriormente, organizarlos y codificarlos por temas y subtemas de análisis lógico.

Para interpretar y determinar las repercusiones de la estructura teórica, se siguieron los pasos de triangulación de Streubert^(17)^, que consisten en reflexionar sobre los hallazgos combinados para extraer conclusiones sobre la calidad de la atención a los pacientes con ACV y el impacto que tiene sobre las experiencias y los resultados de los participantes; identificar oportunidades para mejorar la calidad en función de los conocimientos adquiridos tanto de la investigación fenomenológica como de la estructura de Donabedian; considerar las repercusiones de los hallazgos para las políticas, la práctica, la educación y la investigación en salud. Siguiendo el modelo de calidad de atención de Donabedian^(11)^, el modelo de atención de enfermería de Antunez Martínez y los hallazgos de este estudio^(18)^, se planificó crear una estructura teórica para la atención de enfermería al paciente con ACV.

### Rigor del estudio

El proyecto de investigación aplicó los siguientes criterios de confiabilidad, que consisten en credibilidad, adecuación, auditabilidad y confirmabilidad^(19-20)^. Para cumplir con estos elementos, se aplicaron las estrategias se describen a continuación. Para la credibilidad se utilizó la estrategia de casos negativos, que consiste en comparar las diferentes narrativas con otros elementos en discusión en la búsqueda de datos que estén adecuadamente fundamentados, que parezcan contradictorios o que expliquen los datos emergentes.

Asimismo, se aplicó la estrategia de verificación de miembros como estrategia de confirmabilidad, que consiste en que las interpretaciones, temas y conclusiones sean verificadas por los miembros donde se obtuvieron originalmente los datos. Para la auditabilidad se utiliza la estrategia de registro de auditoría, que consiste en conservar registros y notas relacionadas con la recolección de datos durante los próximos cinco años, además, cabe mencionar que el proceso de investigación contó con diferentes comités de expertos que monitorearon el desarrollo del estudio. Para la adecuación se siguen criterios de flexibilidad, que incluyen la participación de diferentes investigadores con experiencia en el desarrollo de estudios cualitativos desde diferentes enfoques, lo que permite una presentación razonable de los datos después de las discusiones y de lograr el consenso con los involucrados.

### Aspectos éticos

El proyecto fue aprobado por el comité de ética en investigación biomédica de la Facultad de Ciencias Médicas de la Universidad Nacional Autónoma de Honduras (IRB 00003070; código de aprobación 027-2022), y se otorgó la autorización al Departamento de Enfermería del HGA. Este último dio autorización por escrito para realizar la recolección de datos y posteriormente los participantes firmaron el consentimiento libre e informado luego de que el entrevistador les explicara el propósito del estudio y que su participación en el estudio no pondría en riesgo su integridad. Por lo tanto, las identidades de los participantes fueron codificadas para protegerla, utilizando la letra P (participante) seguida del número de entrevista.

## Resultados

Participaron en total 20 enfermeros generales como informantes clave durante la recolección de datos. Sus edades oscilaban entre 24 y 55 años; 10 eran solteros, 5 casados, 2 viudos y 3 tenían uniones registradas; 4 eran hombres y 16 mujeres. Además, en cuanto al nivel de escolaridad, todos los participantes eran enfermeros, sin título de posgrado ni de especialización. Además, su período de experiencia profesional en los departamentos seleccionados era de 1 a 19 años.

Discutimos nuestros hallazgos en tres temas principales, 10 subtemas y 21 conceptos que fueron más abordados por los participantes al reflexionar sobre sus percepciones sobre la calidad de la atención que brinda el equipo de enfermería a los pacientes diagnosticados con ECV en los departamentos de emergencias y clínica médica del HGA ^(Figura 1)^. A continuación, se presentan los grupos temáticos con sus respectivas interpretaciones y citas. Las definiciones de temas y subtemas están disponibles en la Figura 2.

**Figura 1:** - Resumen de los grupos temáticos de las percepciones de enfermeros hondureños sobre la calidad de la atención a pacientes con ACV

**Temas**	**Subtemas**	**Conceptos**
1. Resultados negativos con respecto al tamaño de la estructura de las unidades de atención al paciente	1.1 Espacios insalubres	1.1.1 Desinfección simultánea ambígua 1.1.2 Limpieza esporádica 1.1.3 Personal de limpieza insuficiente
	1.2 Sistema de ventilación inadecuado	1.2.1 Aire acondicionado defectuoso 1.2.2 Falta de ventanas 1.2.3 Contaminación acústica
	1.3 Espacio físico limitado	1.3.1 Sobrepoblación 1.3.2 Privacidad vulnerable
2. Desafíos relacionados con los procesos de atención de enfermería	2.1 Mantener la privacidad del paciente mientras se realizan intervenciones de enfermería	2.1.1 Falta de cooperación de los familiares 2.1.2 Falta de divisores portátiles
	2.2 Falta de suministros y equipamiento	
	2.3 Falta de personal de enfermería especializado	2.3.1 Falta de autonomía profesional 2.3.2 Competencias básicas de atención
	2.4 Personal de enfermería insuficiente en turnos rotativos	2.4.1 Turnos cubiertos por estudiantes de la carrera auxiliar de enfermeira 2.4.2 Sobrecarga de trabajo
	2.5 Ayuda ambigua de otros departamentos del hospital	2.5.1 Diagnósticos atrasados 2.5.2 Tratamientos prolongados 2.5.3 Estancia prolongada del paciente
3. Resultados positivos con respecto al proceso de enfermería	3.1 Atención de enfermería hondureña con ética y humanizada	3.1.1 Empatía 3.1.2 Apoyo emocional 3.1.3 Vocación professional 3.1.4 Compromiso laboral
	3.2 Planificación del alta incluyendo el autocuidado	

**Figura 2:** - Definiciones de los grupos temáticos en función de las percepciones de los enfermeros hondureñas sobre la calidad de la atención al paciente con ACV

GT*	**D** ^†^
1. Resultados negativos con respecto al tamaño de la estructura de las unidades de atención al paciente	No mejorar o mantener la calidad de la atención de enfermería a los pacientes hondureños con ACV ^‡^ en función de la estructura del área donde se encuentran hospitalizados los pacientes.
1.1 Espacios insalubres	Los espacios intrahospitalarios no cumplen con los protocolos de desinfección terminal y simultáneo por parte del personal capacitado.
1.2 Sistema de ventilación inadecuado	Unidades de pacientes con sistemas de intercambio de aire deficientes debido a dispositivos en malas condiciones o ruidosos y sin entradas de aire fresco.
1.3 Espacio físico limitado	Tamaño inadecuado de las unidades para la cantidad de pacientes hospitalizados.
2. Desafíos relacionados con los procesos de atención de enfermería	Cuestionar la validez de los procesos de atención de enfermería con alto nivel de calidad para pacientes con ACV ^‡^ en el sistema público de salud de un país de medio y bajo costo.
2.1 Mantener la privacidad del paciente mientras se realizan intervenciones de enfermería	Dificultades para mantener la seguridad y confidencialidad del paciente durante los procesos de atención complejos debido a la sobrepoblación y la falta de suministros de protección.
2.2 Falta de suministros y equipamiento	No hay o faltan algunos suministros y equipamiento necesarios para los procesos de tratamiento de pacientes con ACV ^‡^ , lo que supone un riesgo para la recuperación exitosa del paciente.
2.3 Falta de personal de enfermería especializado	Los enfermeros no cuentan con la formación adecuada para el cuidado de pacientes con ACV ^‡^ , por lo tanto, tienen malas experiencias laborales y posibles niveles más altos de estrés relacionado con el trabajo y los bajos niveles de seguridad del paciente.
2.4 Personal de enfermería insuficiente en turnos rotativos	No se contrata suficiente personal para las áreas críticas y la intensidad de la carga de trabajo es demasiado alta como para gestionarla de forma segura en los diferentes turnos.
2.5 Ayuda ambigua de otros departamentos del hospital	Un estado de incertidumbre intencional, patrón de comportamiento o situación que puede malinterpretarse debido a la resistencia que tiene a cumplir con su responsabilidad el personal de los departamentos de laboratorio, imágenes y radiación. Por lo tanto, eso afecta el diagnóstico y los planes de tratamiento del paciente, y aumenta la duración de la estancia hospitalaria.
3. Resultados positivos con respecto al proceso de enfermería	Experiencias placenteras y útiles para los procesos de enfermería en el cuidado de pacientes con ACV ^‡^ .
3.1 Atención de enfermería hondureña ética y humanizada	Considerar el estado de salud y los sentimientos del paciente y sus familiares para ajustar su comportamiento y atención para demostrar que el sistema de salud es razonable y flexible. Por lo tanto, la atención de enfermería hondureña no se basa en intenciones institucionales, sino en actitudes orientadas al bienestar del paciente.
3.2 Planificación del alta incluyendo el autocuidado	Alta formal del paciente del hospital cuando presente mejor estado de salud y pueda recibir tratamiento ambulatorio en su domicilio, con la participación de la familia en los planes de cuidados. Las actividades de promoción del autocuidado y preparación de los familiares se realizan durante todo el período de internación y son responsabilidad del equipo de enfermería.

*GT = Grupos Temáticos; ^†^D = Definiciones; ^‡^ACV = Accidente cerebrovascular

### Resultados negativos con respecto al tamaño de la estructura de las unidades de atención al paciente

Espacios insalubres: Los participantes informan que los servicios son inadecuados, ya que no cuentan con un proceso de desinfección adecuado y frecuente. Es decir, no cumplen con el número de desinfecciones simultáneas y terminales que requiere la complejidad de los servicios de emergencia y clínica médica. Esto ocurre porque el personal de limpieza no está asignado a permanecer en los departamentos y, además, realiza la desinfección simultánea de lunes a viernes, una vez al día. Mientras que el resto de los días, son los familiares de los pacientes quienes realizan dichas actividades. Esto compromete la calidad de las instalaciones, porque los familiares de los pacientes no cuentan con la capacitación técnica para realizar las actividades de desinfección adecuadas. Cabe señalar que no está documentada la frecuencia de las desinfecciones terminales en la instalación.

*Falta higiene... el personal de limpieza limpia, pero no a fondo. Como estamos en una sala de emergencias, apenas llega un paciente con un evento cerebrovascular, llegan otros enfermos renales y manchan las paredes, las camas, y no solo eso, sino que también queda sangre en el piso.* (P1)

*Eso no está bien. Porque no vienen a limpiar todos los días ni de vez en cuando, sino cuando tienen tiempo... debería haber un equipo específico para el área de emergencia y aquí no vi ninguno.* (P3)

*El personal de limpieza solo trabaja por la mañana, de lunes a viernes... los mismos familiares nos ayudan con la desinfección.* (P14)

*Hay solo un equipo de limpieza y tiene que limpiar más áreas.* (P17)

Sistema de ventilación inadecuado: Los servicios no cuentan con un sistema de ventilación adecuado, dado que los empleados informan que no pueden abrir la única ventana disponible por el riesgo de entrada de vectores infecciosos externos al hospital. Los sistemas de ventilación como ventiladores y aires acondicionados no están en buenas condiciones, por lo que las familias de los pacientes deben traer sus propios suministros para mejorar las condiciones de vida de los pacientes. Cabe destacar que no utilizan los ventiladores que se encuentran en los servicios porque esto implica un riesgo de contaminación acústica y desarmonización del ambiente debido al ruido que producen los mismos cuando están en funcionamiento.

*La estructura es deplorable porque no tenemos buena ventilación. Las unidades de aire acondicionado no funcionan... hace muchísimo calor. Esto afecta el desempeño del personal... hay ventanas, pero no se abren porque como estamos atrapados del otro lado pueden entrar insectos, o cuando llueve, ese mismo viento trae agua.* (P1)

*La sala no está bien acondicionada... y eso afecta a los pacientes porque algunos se sofocan, sienten mucho calor, incluso para nosotras las enfermeras... el área no está ventilada.* (P7)

*Cada paciente trae su propio ventilador... pero no es suficiente, y nuestro ventilador también hace mucho ruido. Usarlo genera contaminación acústica.* (P13)

Espacio físico limitado: Los participantes informaron que las dimensiones de los cubículos, al igual que la cantidad, son limitadas; Esto se debe a que no son proporcionales al número de pacientes que recibe atención en el servicio. Por lo tanto, cuando el ingreso de pacientes es elevado, el equipo de enfermería se ve obligado a colocar a varios pacientes en un mismo cubículo, sin respetar la distancia adecuada entre ellos. Esta situación es alarmante por el mayor riesgo de infecciones intrahospitalarias.

*Las dimensiones de los cubículos y la cantidad de unidades disponibles no son las adecuadas, ... no solo recibimos pacientes con ACV, sino también con sospecha de COVID-19, que es la enfermedad más común hoy en día, y no podemos respetar el distanciamiento adecuado para evitar infecciones hospitalarias.* (P1)

*En cada cubículo se colocan más pacientes de los necesarios, y esto se debe al alto número de pacientes ingresados... Los cubículos son muy pequeños en relación al número de pacientes ingresados. No hay espacio... a veces por las condiciones de los cubículos, en lugar de ayudar al paciente, complica aún más su condición.* (P8)

### Desafíos relacionados con los procesos de atención de enfermería

Mantener la privacidad del paciente durante las intervenciones de enfermería: Este es uno de los desafíos que enfrenta el equipo de enfermería cuando atiende a pacientes con ACV en los departamentos de emergencias y clínica médica. Las condiciones de los servicios no son favorables para la alta demanda de pacientes, por lo que el personal se ve obligado a ubicar a varios pacientes en un mismo cubículo en diferentes unidades muy cerca unos de otros. Los familiares y otros visitantes insisten en permanecer en la unidad y el equipo de enfermería no cuenta con biombos para proteger la privacidad de los pacientes, lo que afecta la calidad de los procesos de atención de enfermería.

*En algunos casos tenemos que realizar procedimientos en privado para preservar la integridad del paciente, pero los familiares no quieren salir del cubículo... en la otra unidad, en el mismo cubículo, hay otros pacientes con sus familias, y tenemos que encontrar la forma de preservar la privacidad de los pacientes, aunque no siempre sea posible*. (P4)

*El problema son los familiares, aunque les digamos que es una zona restringida, siempre hay visitas y familiares con el paciente.* (P5)

*Actualmente, no hay biombos y hay varios pacientes y familiares en un mismo cubículo.* (P9)

Falta de suministros y equipamiento: Los departamentos carecen de equipamiento y suministros médico-quirúrgicos para brindar atención de enfermería de calidad a los pacientes con accidente cerebrovascular. El hospital cubre algunas necesidades básicas; sin embargo, la cantidad no coincide con el número de pacientes que recibe atención en los departamentos. Además, en lo que respecta al equipamiento médico-quirúrgico, los departamentos no cuentan con suficiente, por lo que el personal de enfermería se ve obligado a desplazarse a otros servicios para tomar prestado su equipamiento. Esto implica una pérdida de tiempo y energía, sin mencionar que otros departamentos se quedan temporalmente sin el equipamiento necesario para atender a los pacientes críticos.

*Faltan suministros. Muchas veces ni siquiera disponemos de elementos básicos como jeringas, catéteres intravenosos, soluciones intravenosas... La administración del hospital nos vuelve a dar, pero no lo suficiente... y no tenemos todo el material médico-quirúrgico que necesitan los pacientes con AVC... tenemos que pedirles a otros departamentos que nos presten su equipamiento. Perdemos tiempo trasladándonos.* (P1)

*Deberíamos contar con más equipamiento y suministros médico-quirúrgicos para brindar una mejor atención a los pacientes con ACV. Para mí es un desafío trabajar en estas condiciones... faltan insumos para brindar un buen servicio.* (P3)

*Lo que hacemos es darle la receta a los familiares para que compren lo que necesitan.* (P12)

Falta de personal de enfermería especializado: Los departamentos de clínica médica y de emergencia no cuentan con personal de enfermería especializado en cuidados críticos y de emergencia, sus competencias se limitan a los conocimientos básicos de atención y la mayoría del personal no tiene experiencia clínica. Por ende, la falta de personal especializado hace que los enfermeros del departamento dependan de otras disciplinas para tomar decisiones sobre la atención del paciente con ACV, y pierden su autonomía profesional y liderazgo, lo que compromete la calidad de la atención en la dimensión del proceso.

*El médico especialista se encarga de varios procedimientos complejos porque no hay enfermeros especialistas, y eso es un problema... porque yo, por ejemplo, no le presto tanta atención al ventilador mecánico porque, aunque tengo conocimientos básicos, siempre debe haber un enfermero especialista que atienda a los pacientes con ACV y no hay ninguno en el departamento.* (P4)

*El personal de enfermería del departamento no es especialista y no tiene experiencia, por lo que no comprende la complejidad de un paciente con ACV. Lo que compromete la calidad del servicio, las tareas simplemente se realizan.* (P5)

*Estos pacientes con ACV necesitan atención especializada... Solo tenemos enfermeros generales y no todos están capacitados profesionalmente porque son empleados nuevos... hay pacientes que necesitan atención que solo los enfermeros especializados pueden brindar. Entonces nos volvemos dependientes de los médicos para estos procesos. El enfermero pierde autonomía como profesional.* (P7)

Personal de enfermería insuficiente en turnos rotativos: Un desafío importante en los departamentos de emergencias y clínica médica es la falta de personal de enfermería para cada turno. Los participantes informan que hay turnos en los que solo hay una enfermera general responsable de 14 pacientes críticos y, en ocasiones, necesita compartir sus responsabilidades con estudiantes y auxiliares de enfermería porque ella sola no puede cubrir todas las necesidades de los pacientes. Sin embargo, quedan cosas pendientes para los próximos turnos que enfrentan el mismo problema, lo que compromete la calidad de la atención a los pacientes con ECV.

*Los alumnos de la carrera de auxiliar de enfermería colaboran, realizan algunos turnos con nosotros.* (P2)

*Hay falta de personal. A veces solo hay un auxiliar de enfermería, un estudiante de enfermería y un enfermero general... cuando hay empleados de vacaciones o incapacitados, solo queda una persona por turno.* (P6)

*En los turnos de tarde, la rotación de pacientes es muy difícil. A veces estoy sola como enfermera, tal vez pueda recibir ayuda de algún familiar, pero sigue siendo complicado.* (P13)

*A veces hay 13 pacientes por enfermera, y me refiero a un departamento que atiende a pacientes en condiciones críticas. Por eso no es posible atender a tantos pacientes... hay muchas cosas pendientes.* (P14)

*Necesitamos más personal, ya que no solo tenemos pacientes con ACV admitidos para observación, sino que también atendemos a otros pacientes que llegan por otras emergencias.* (P19)

Ayuda ambigua de otros departamentos del hospital: Los participantes describen dificultades en el proceso de diagnóstico y tratamiento de pacientes con ECV debido a la ayuda ambigua de los departamentos de apoyo. El equipo de imágenes no realiza todos los procedimientos diagnósticos necesarios, además tiene horarios de atención restringidos. A su vez, el equipo de laboratorio dice que no tiene disponibilidad para proporcionar en tiempo y forma los resultados de las pruebas solicitadas. Por lo tanto, el personal de enfermería de los departamentos de emergencias y clínica médica debe invertir tiempo y esfuerzo en facilitar los procesos del departamento de apoyo para obtener los servicios solicitados.

*A veces se demora mucho en detectar el tipo de ECV que tiene el paciente... Entonces nos atrasamos... no se hacen radiografías todos los días, tienen un horario, pero a veces vienen y dicen que estará disponible hasta las 18 hs.; A veces vienen y dicen: hoy no vamos a hacer radiografías ni tomografías.* (P5)

*El departamento de laboratorio falla al 100% y dependemos de ellos... Se demoran mucho en entregar los resultados, a pesar de que les ayudamos a recolectar las muestras [...] el departamento de farmacia solo nos da medicamentos de uso diarios según la prescripción, pero si tenemos una emergencia dependemos de si el personal quiere dárnosla o no. Perdemos mucho tiempo en eso.* (P7)

### Resultados positivos con respecto al proceso de enfermería

Atención de enfermería hondureña ética y humanizada: Entre los hallazgos positivos del estudio en la dimensión proceso se encuentra el trato humanizado con ética profesional por parte del equipo de enfermería a los pacientes con ECV en los servicios de emergencia y clínica médica. Este trato se basa en la empatía, dado que considera los sentimientos de los pacientes. Transmitir energía positiva al paciente, al comportarse de forma alentadora, brindarle apoyo emocional y manteniendo abiertos los canales de comunicación tanto con el paciente como con sus familiares. Este tipo de conducta está presente en el equipo de enfermería comprometido con la recuperación del paciente y con vocación por su profesión.

*Empatía... Si el paciente está consciente y nos mira con mala cara, es porque se siente mal. Por eso, siempre intentamos estar alegres y compartir energía positiva con el paciente.* (P1)

*Soy una persona que trata a los familiares con amabilidad... Intento tratar al paciente como me gustaría que trataran a mi familia... Me gustaría que me vieran de la misma manera que miro a los pacientes*. (P4)

*Los pacientes con ACV son vulnerables, por eso tenemos que prestar atención y darles el máximo apoyo posible, hablar con ellos... animarlos, ... actuar con empatía... darles apoyo emocional... necesitan sentir la empatía de los enfermeros*. (P12)

*Me siento bien porque elegí esta profesión que me gusta. Por eso, atiendo a los pacientes con ACV con la mejor actitud.* (P14)

Planificación del alta incluyendo el autocuidado: El proceso de planificación del alta del paciente del servicio de hospitalización para el retorno seguro y autónomo del mismo a su domicilio, incluyendo a la familia en las actividades de cuidado. En Honduras, el enfermero es responsable de las actividades educativas y administrativas relacionadas con el alta del paciente.

*El paciente y sus familiares reciben información sobre la atención que necesitan los pacientes con ACV... nos tomamos el tiempo para informarles qué hacer cuando regresen a casa.* (P6)

Al fusionar la investigación fenomenológica con la teoría de Donabedian sobre la calidad de la atención para pacientes con ACV, obtuvimos una comprensión profunda de las experiencias de los pacientes y los factores que afectan la calidad de la atención médica. A través de la triangulación, identificamos patrones, temas e inconsistencias en los datos, lo que generó sugerencias para mejorar la calidad de la atención a los pacientes con ACV. Comparar y combinar los hallazgos de la investigación fenomenológica, la estructura de Donabedian y el modelo de atención de enfermería de Antunez Martínez nos permitió comprender la calidad de la atención a los pacientes con ACV, tanto desde la perspectiva de los participantes como desde el punto de vista de mejorar la calidad de la atención ^(Figura 3)^.

**Figura 3:** - Estructura teórica para la calidad de la atención de enfermería al paciente con ACV

Nota: Elaborado por los autores en base a los resultados del estudio, al modelo de calidad de atención de Donabedian y al modelo de atención de enfermería de Antunez Martínez

## Discusión

En este estudio se obtuvieron tres temas: Resultados negativos con respecto al tamaño de la estructura de las unidades de atención al paciente, Desafíos relacionados con los procesos de atención de enfermería y Resultados positivos con respecto al proceso de enfermería. Este estudio, realizado con una muestra de enfermeros hondureños de salas de emergencia y clínica médica, coincide con los resultados de estudios de países de altos ingresos que exploran las experiencias del personal de salud sobre la calidad de la atención a los pacientes con ACV.

Los pacientes con ACV requieren atención especializada, así como el equipamiento necesario para un diagnóstico rápido, eficaz y oportuno. Sin embargo, los servicios de emergencia y clínica médica de Honduras no cuentan con todo el equipamiento necesario para brindar la atención que requieren los pacientes con accidente cerebrovascular, lo que provoca un retraso en el diagnóstico de este tipo de pacientes, según el tipo (hemorrágico o isquémico). Pero, el proceso de atención de enfermería debe continuar. Estas deficiencias afectan directamente la calidad de la atención a los pacientes con este diagnóstico, tanto por la falta de equipamiento como por la falta de personal de enfermería competente para la prestación de cuidados.

El hallazgo anterior coincide con el de un estudio realizado en el norte de Inglaterra, donde los resultados resaltan que la falta de recursos y del medio ambiente actúan como barrera para brindar una atención de calidad e implementar directrices de intervención^(21)^. Cabe destacar que la mayoría de los estudios sobre la calidad de la atención son de países desarrollados y, por lo tanto, su foco está en la organización del personal y sus competencias específicas, y no en la falta de insumos y de espacios adecuados para la hospitalización^(22-23)^. A excepción de un estudio realizado en Suecia, que destaca cuestiones ambientales, la investigación no aborda las necesidades de atención específicas de los pacientes con accidente cerebrovascular^(24)^. Se recomienda continuar estudiando este tema en los sistemas de salud de los países de medianos y bajos ingresos.

En cuanto a la dimensión del proceso, los participantes informaron que en el servicio se llevaban a cabo la atención integral y competencias generales de enfermería. Por ende, algunos procedimientos complejos son realizados por el equipo médico, lo que implica una pérdida de espacio para el personal de enfermería como profesionales autónomos y líderes de los procesos asistenciales.

Para realizar la atención de los pacientes con ACV antes mencionados^(25)^, es necesario contar con grandes habilidades y competencias clínicas para interactuar con el paciente y sus familiares. Por lo tanto, la formación continua es relevante para desarrollar la competencia de los profesionales de la salud para brindar atención de enfermería especializada como parte de una atención de calidad adecuada^(26-28)^.

Un estudio transversal realizado en Inglaterra, Bélgica, Finlandia, España, Irlanda, Islandia y Suiza muestra que la reducción de responsabilidades clínicas complejas del equipo de enfermería y la sobrecarga del equipo médico pueden contribuir a que haya muertes evitables, lo que compromete la calidad y seguridad de la atención hospitalaria^(29)^. Es necesario realizar estudios que exploren la dinámica de la organización del trabajo de enfermería con otros profesionales de la salud para comprender este fenómeno, así como también estudios cuantitativos para medir y describir las competencias de los enfermeros en el sistema de salud pública latinoamericano. Posteriormente, se recomienda crear políticas públicas que garanticen más espacios para que el personal de enfermería lleve a cabo intervenciones avanzadas, y promover las competencias especializadas en la atención crítica del paciente con ACV.

Además, en las narrativas de los participantes se destaca la ayuda ambigua de otros servicios, como los de laboratorio y de rayos X. Este hecho es consistente con los resultados de otros estudios que destacan que la respuesta tardía de los servicios de apoyo al diagnóstico y tratamiento del ACV es una barrera para brindar una atención de calidad^(30-32)^. Este problema, al igual que el detectado en nuestro estudio, se debe a la resistencia del equipo de salud a cambiar de actitud y al número limitado de empleados.

El fenómeno anterior no sigue los principios del modelo de Donabedian^(11)^, que establece que la evaluación de la calidad del proceso es el conjunto de acciones que hay que realizar para lograr un resultado específico. Es un proceso complejo de interacción o comunicación entre el paciente y el equipo de salud, además de la tecnología utilizada, las habilidades especializadas que debe tener el equipo de salud para atender a este tipo de pacientes y la ejecución oportuna de los procedimientos de diagnóstico y tratamiento del paciente, que deben jugar un papel importante.

Entre los hallazgos positivos se destacan la postura ética profesional y el trato humanizado del equipo de enfermería al paciente con ACV. Además, esos pacientes requieren mejor acceso al apoyo emocional que puede brindar el equipo de salud, que incluye información y asesoramiento tanto para el paciente como para sus familiares, lo cual es congruente con el accionar de los enfermeros hondureños enfocados en el trato humanizado y el fortalecimiento del autocuidado durante el proceso de alta^(32-34)^. Es necesario que se realicen más investigaciones para establecer la eficacia del apoyo emocional y las competencias de salud mental de los enfermeros.

La importancia de la educación del paciente y su familia durante el alta la destaca un autor que menciona que el proceso de recuperación después de un ACV es complejo y tedioso, por lo que es necesario contar con una buena orientación sobre los próximos pasos a seguir^(35)^. Los participantes de este estudio priorizan esa actividad educativa. También se recomienda explorar dichas actividades y el protocolo establecido para el alta del paciente con ACV desde la perspectiva de la enfermería.

Las limitaciones incluyeron el hecho de que el personal de enfermería que trabajaba en los departamentos de clínica médica y de emergencia del HGA no estaba acostumbrado a participar en este tipo de enfoque de investigación; eso les provocó cierto estrés porque la entrevista fue completamente grabada, lo que, en ocasiones, limitó la manifestación espontánea y completa de las respuestas a las preguntas formuladas.

Además, el nivel de preparación de los participantes fue una limitación. Se considera que el desarrollo de esta metodología con enfermeros de emergencias y cuidados intensivos puede contribuir al desarrollo de un modelo ampliado de práctica de enfermería para la atención de pacientes con ACV. Por último, el estudio se realizó en una sola localidad, lo impide tener una visión más general del fenómeno en Honduras.

Entre las fortalezas se considera el número de participantes, que permitió aumentar la representatividad del estudio. Además, la persona responsable de realizar las entrevistas fue una enfermera con experiencia en estudios cualitativos que trabaja en la misma institución, lo que facilitó que los profesionales contactados aceptaran participar del estudio.

## Conclusión

Las experiencias de los enfermeros hondureños revelaron que el escenario para los pacientes con ACV en el sector de la salud era desafiante. La infraestructura existente no puede cumplir con los estándares básicos de calidad, lo que pone en riesgo la seguridad del paciente. Esto arroja luz sobre la realidad de los servicios hospitalarios en Honduras y las exigentes condiciones laborales que enfrentan los enfermeros, que a menudo se pasan por alto, pero que aportan ideas aplicables para mejorar la situación en todo el mundo. Los enfermeros de los servicios de emergencia y clínica de Honduras no cuentan con capacitación especializada en cuidados críticos, eso representa una limitación que afecta la calidad de la atención que brindan, dada la complejidad de las necesidades de los pacientes con ACV. Es necesario garantizar urgentemente que los departamentos de emergencia cuenten con personas capacitadas para liderar los procesos de atención de manera efectiva. A pesar de estos desafíos, surge un hecho positivo en la dimensión humanización y tratamiento ético, pues el equipo de enfermería demuestra que brinda apoyo y siente empatía, esos son factores cruciales que merecen consideración.

Al triangular la investigación fenomenológica con la teoría de Donabedian, es posible obtener una comprensión más profunda de la compleja interacción entre las experiencias de los enfermeros y la calidad de la atención médica en el contexto de la atención del ACV, lo que en última instancia conduce a intervenciones más informadas y efectivas para mejorar los resultados y la satisfacción de los pacientes. Quienes formulan políticas y gestionan la atención médica deben reconocer que es necesario incorporar enfermeros con habilidades avanzadas al proceso de atención para garantizar la recuperación del usuario con el más alto nivel de atención de enfermería de calidad en los países de medianos y bajos ingresos. Al igual que contar con estructuras organizativas claras y una mejor gestión de los recursos. Este tipo de estrategias traerán mayores beneficios a las instituciones de salud pública y aumentarán la satisfacción de los usuarios, así como reducir las estancias hospitalarias.

# Translation: Percepções de enfermeiros hondurenhos sobre a qualidade do atendimento a pacientes com AVC: um estudo qualitativo

**DOI:** 10.1590/1518-8345.7300.4420

**Authors:** Sandra Patricia Nájera Figueroa, Oscar Fidel Antunez Martinez, Perla Simons Morales

**Affiliations:** 1 National Autonomous University of Honduras, School of Nursing, Tegucigalpa, Francisco Morazán, Honduras.; 2 National Taipei University of Nursing and Health Sciences, College of Nursing, Beitou District, Taipei, Taiwan

**Keywords:** Enfermagem em Emergência, Enfermagem Cardiovascular, Acidente Vascular Cerebral, Qualidade da Assistência à Saúde, Honduras, Pesquisa Qualitativa

## Abstract

**Objetivo:**

explorar as percepções dos enfermeiros sobre a qualidade do atendimento a pacientes com AVC em um hospital público no norte de Honduras.

**Método:**

foi realizado um estudo fenomenológico descritivo. A coleta de dados foi realizada por meio de entrevistas em profundidade com 20 enfermeiras gerais dos departamentos de emergência e medicina clínica do Hospital Geral de Atlántida. A análise dos dados foi feita por meio da técnica temática.

**Resultados:**

a pesquisa encontrou três temas principais e 10 subtemas que ilustraram a qualidade do atendimento a pacientes com AVC em um hospital geral de Honduras. A descoberta aponta para o tema “resultados negativos em relação à dimensão da estrutura das unidades de atendimento ao paciente”, que significa a falha em melhorar ou manter a qualidade da assistência médica. Da mesma forma, “resultados positivos em relação ao processo de enfermagem”, definidos como agradáveis e úteis no atendimento a pacientes com AVC, como o cuidado ético e humanizado e as atividades de promoção do autocuidado do paciente com o envolvimento dos familiares.

**Conclusão:**

os resultados indicam que os enfermeiros hondurenhos não têm treinamento qualificado de pós-graduação em cuidados críticos, o que é uma limitação que compromete a qualidade do atendimento. Portanto, recomenda-se ter estruturas organizacionais claras e melhor gerenciamento de recursos, o que, consequentemente, pode aumentar a satisfação do usuário e reduzir o tempo de permanência no hospital.

## Destaques

**(1)** Os departamentos de emergência hondurenhos não contam com enfermeiros competentes em cuidados críticos.

**(2)** O atendimento humanizado aumenta a satisfação do paciente.

**(3)** A equipe de enfermagem está sobrecarregada devido à falta de apoio de outros departamentos.

**(4)** Sugere-se uma estrutura para a qualidade do atendimento de enfermagem a pacientes com AVC.

**(5)** Um desafio no atendimento ao paciente com AVC é a falta de equipe de enfermagem para cada turno.

## Introdução

Uma doença cerebrovascular (DCV) ou acidente vascular cerebral (AVC) é um comprometimento agudo da perfusão cerebral ou da vasculatura^(1)^. Nas últimas décadas, a incidência de AVC e a mortalidade estão diminuindo; no entanto, o AVC é a principal causa de incapacidade em adultos em todo o mundo^(1)^. Dada a sua contribuição substancial para os custos crescentes da assistência médica, o AVC também gera uma alta carga socioeconômica na população em geral, o que destaca a importância da pesquisa de enfermagem em relação ao gerenciamento e à qualidade da assistência^(2)^. A qualidade do atendimento consiste em realizar cada uma das atividades voltadas para o paciente por meio da aplicação da ciência e da tecnologia de forma a maximizar seus benefícios à saúde sem aumentar proporcionalmente seus riscos^(3-5)^.

Os serviços de saúde na América Latina sofrem com a falta de orçamentos, o que limita a qualidade do atendimento e a disponibilidade de recursos, afetando a satisfação do usuário; consequentemente, os modelos de gestão desses serviços diferem de acordo com o contexto socioeconômico e cultural em que são desenvolvidos. Da mesma forma, o pessoal que presta esses serviços enfrenta muitas restrições, principalmente nos países em desenvolvimento, onde o desempenho do pessoal é medido pela prestação de serviços a um número maior de pacientes, em detrimento da qualidade do atendimento^(6-8)^.

Em Honduras, as instalações de saúde onde o atendimento a pacientes com AVC é prestado por uma equipe de enfermagem com diploma técnico e habilidades técnicas, assim como por enfermeiros gerais com apenas graduação que desempenham várias funções, são limitadas no momento do desenvolvimento de suas atividades devido à escassez de equipamentos, suprimentos e recursos humanos em consequência dos baixos orçamentos disponíveis nos hospitais públicos^(8-9)^. Deve-se observar que o atendimento a esses pacientes não é protocolizado, não há planos de cuidados orientados para essa patologia e não há publicações com o escopo da enfermagem hondurenha, o que impede a prestação de cuidados de forma equitativa, lógica e sistemática para oferecer, na medida do possível, uma melhor qualidade de atendimento para o manejo de pacientes com essa doença. Permanecem lacunas significativas no tratamento e no cuidado das DCVs, portanto, esse fenômeno precisa ser explorado com uma abordagem qualitativa para conhecer as experiências e as percepções da enfermagem no cuidado dos pacientes com AVC.

A avaliação da qualidade da assistência médica deve ser fundamentada em uma definição conceitual e operacional clara do que significa qualidade da assistência para os enfermeiros^(10)^. Normalmente, a eficácia do atendimento médico, medida pelas taxas de recuperação, restauração funcional e sobrevivência, é usada como um indicador da qualidade do atendimento^(10)^. Além disso, o modelo de Donabedian propôs três abordagens para avaliar a qualidade do atendimento: estrutura, processo e resultado^(11)^. Neste estudo, a ênfase foi dada à estrutura e ao processo, uma vez que a abordagem qualitativa permite explorar as percepções dos participantes sobre esses elementos e não busca quantificar os resultados. Vale ressaltar que Donabedian faz um julgamento sobre uma frase: uma boa estrutura aumenta a possibilidade de um bom processo e, consequentemente, aumenta a possibilidade de um bom resultado^(12-14)^.

Assim, o objetivo geral deste estudo foi explorar as percepções dos enfermeiros hondurenhos sobre a qualidade do atendimento a pacientes com AVC em um hospital público no norte de Honduras. Espera-se que os resultados deste estudo contribuam para a formulação de políticas e para o desenvolvimento de protocolos de atendimento em relação à atenção dos enfermeiros aos pacientes com AVC.

## Método

### Desenho e cenário do estudo

Este estudo fenomenológico descritivo teve como objetivo explorar as percepções da equipe de enfermagem em relação à qualidade do atendimento prestado aos pacientes com DCV nos departamentos de Emergência e Medicina Clínica do Hospital Geral de Atlántida (HGA), La Ceiba, Honduras, seguido de análise temática. Esse tipo de abordagem oferece uma maneira nova e inovadora de entender as experiências individuais e fornece evidências para a proposição de soluções baseadas em evidências, design de estrutura, que podem melhorar a qualidade da assistência de enfermagem.

### Recrutamento, critérios de seleção e definição da amostra

Os participantes eram um total de 20 enfermeiros gerais dos departamentos de emergência e de medicina clínica do HGA, Atlántida, Honduras. O estudo usou uma técnica de amostragem intencional para recrutar os participantes, por meio da programação de turnos de enfermagem do departamento de emergência, que foi fornecida pelo diretor do departamento. De acordo com Morse, recomenda-se um mínimo de seis e um máximo de 20 participantes para estudos fenomenológicos. Portanto, as entrevistas ocorreram até que a saturação dos dados fosse atingida^(15)^, o que significa que uma certa diversidade de ideias já foi ouvida em cada entrevista até que outros elementos aparecessem.

Os critérios de inclusão foram: ser um enfermeiro que trabalhasse em diferentes turnos e com pelo menos um ano de experiência no atendimento de pacientes diagnosticados com DCV nos departamentos de emergência ou de medicina clínica do HGA, e não estar de férias ou incapacitado durante a coleta de dados.

### Procedimento e instrumento de coleta de dados

Os dados foram coletados entre agosto e outubro de 2022 no idioma nativo dos participantes (espanhol) por meio de entrevistas em profundidade. O roteiro da entrevista continha cinco perguntas abertas com os principais conceitos do estudo, com duração aproximada de 20 a 45 minutos. As entrevistas foram realizadas em um espaço tranquilo e privado próximo à sala de espera do departamento de emergência, permitindo que os participantes expressassem suas opiniões e emoções confortavelmente. Além disso, o instrumento foi validado por um comitê especializado em metodologia de pesquisa qualitativa e que tem como campo de pesquisa a enfermagem de emergência ou de DCV.

A principal autora do estudo, uma enfermeira que não trabalhava no departamento de emergência, realizou a coleta de dados. No entanto, ela é enfermeira de outro departamento do mesmo hospital e, portanto, está familiarizada com o pessoal. A entrevistadora tem experiência em desenvolver entrevistas semiestruturadas e foi orientada por um especialista em pesquisa qualitativa durante a coleta de dados. Da mesma forma, o estudo foi realizado no departamento de emergência do HGA, por ser uma instituição de saúde de referência por seu nível de complexidade na prestação de atendimento médico especializado gratuito aos casos mais graves do norte de Honduras.

### Tratamento e análise de dados

Ele foi desenvolvido seguindo as sete etapas de Colaizzi, que consistem em familiarização, identificação das principais narrativas, formulação de significados, agrupamento de temas, desenvolvimento de uma descrição exaustiva, produção da estrutura fundamental e busca de verificação da estrutura^(16)^. Primeiramente, os dados obtidos serão transcritos e, em seguida, organizados e codificados por temas e subtemas de análise lógica.

Para a interpretação e as implicações da estrutura teórica, foram seguidas as etapas de triangulação de Streubert^(17)^, que consistem em refletir sobre as descobertas combinadas para tirar conclusões sobre a qualidade do atendimento a pacientes com AVC e seu impacto nas experiências e nos resultados dos participantes; identificar oportunidades de melhoria da qualidade com base nas percepções obtidas tanto da pesquisa fenomenológica quanto da estrutura de Donabedian; considerar as implicações das descobertas para a política, a prática, a educação e a pesquisa em saúde. Seguindo o modelo de Donabedian para a qualidade da assistência^(11)^, o modelo de assistência de enfermagem de Antunez Martinez e as descobertas deste estudo^(18)^, planejou-se criar uma estrutura teórica para a assistência de enfermagem a pacientes com AVC.

### Confiabilidade

O projeto de pesquisa aplicou os seguintes critérios de confiabilidade, que consistem em credibilidade, adequação, auditabilidade e confirmabilidade^(19-20)^. Para atender a esses elementos, foram aplicadas as seguintes estratégias, descritas a seguir. Para a credibilidade, foi usada a estratégia dos casos negativos, que consiste em comparar as diferentes narrativas com outros elementos em discussão na busca de dados que sejam devidamente fundamentados, que pareçam contraditórios ou que expliquem os dados emergentes.

Da mesma forma, aplicou-se a estratégia de verificação dos participantes como estratégia de confirmabilidade, que consiste em que as interpretações, os temas e as conclusões sejam verificados pelos indivíduos que forneceram os dados originalmente. Para a auditabilidade, utiliza-se a estratégia de trilha de auditoria, que consiste em manter as gravações e as anotações relacionadas à coleta de dados pelos próximos cinco anos, e vale a pena mencionar que o processo de pesquisa contou com diferentes comitês de especialistas que acompanharam o desenvolvimento do estudo. Para a adequação, são seguidos os critérios de flexibilidade, que contam com a participação de diferentes pesquisadores com experiência no desenvolvimento de estudos qualitativos a partir de diferentes abordagens, permitindo assim a apresentação razoável dos dados após discussões e consenso com os envolvidos.

### Aspectos éticos

O projeto foi aprovado pelo comitê de ética em pesquisa biomédica da Faculdade de Ciências Médicas da Universidad Nacional Autonóma de Honduras (IRB 00003070; código de aprovação 027-2022), e a autorização foi concedida ao Departamento de Enfermagem do HGA. Esse último deu autorização por escrito para a realização da coleta de dados e, em seguida, os participantes assinaram o consentimento informado depois que o entrevistador explicou a eles o objetivo do estudo e que sua participação no estudo não colocaria sua integridade em risco. Portanto, as identidades dos participantes foram codificadas como um método de proteção, usando a letra P (participante) seguida do número da entrevista.

## Resultados

Um total de 20 enfermeiras gerais participaram como informantes-chave durante a coleta de dados. Suas idades variaram de 24 a 55 anos; 10 eram solteiros, 5 casados, 2 viúvos e 3 uniões registradas; 4 eram homens e 16 mulheres. Além disso, com relação ao nível de escolaridade, todos os participantes eram enfermeiros registrados, sem diploma de pós-graduação ou especialização. Além disso, seu período de experiência profissional foi de 1 a 19 anos nos departamentos selecionados.

Discutimos nossos achados em 3 temas principais, 10 subtemas e 21 conceitos que foram mais levantados pelos participantes ao refletirem sobre suas percepções a respeito da qualidade do atendimento prestado pela equipe de enfermagem a pacientes com diagnóstico de DCV nos departamentos de Emergência e Medicina Clínica do HGA ^(Figura 1)^. A seguir, estão disponíveis os grupos temáticos com suas respectivas interpretações e citações. As definições dos temas e subtemas estão disponíveis na Figura 2.

**Figura 1:** - Resumo dos grupos temáticos das percepções dos enfermeiros hondurenhos sobre a qualidade do atendimento a pacientes com acidente vascular cerebral

**Temas**	**Subtemas**	**Conceitos**
1. Resultados negativos em relação à dimensão da estrutura das unidades de atendimento ao paciente	1.1 Espaços insalubres	1.1.1 Desinfecção simultânea ambígua 1.1.2 Limpeza esporádica 1.1.3 Pessoal de limpeza insuficiente
	1.2 Sistema de ventilação inadequado	1.2.1 Ar-condicionado com defeito 1.2.2 Falta de janelas 1.2.3 Poluição sonora
	1.3 Espaço dimensional limitado	1.3.1 Superlotação 1.3.2 Privacidade vulnerável
2. Desafios relacionados aos processos de assistência de enfermagem	2.1 Manter a privacidade do paciente durante a realização de intervenções de enfermagem	2.1.1 Não cooperação dos familiares 2.1.2 Falta de divisórias portáteis
	2.2 Falta de suprimentos e equipamentos	
	2.3 Falta de pessoal de enfermagem especializado	2.3.1 Falta de autonomia profissional 2.3.2 Competências básicas de atendimento
	2.4 Equipe de enfermagem insuficiente em turnos rotativos	2.4.1 Turnos cobertos por alunos assistentes de enfermagem 2.4.2 Sobrecarga de trabalho
	2.5 Apoio ambíguo de outros departamentos do hospital	2.5.1 Diagnósticos atrasados 2.5.2 Tratamentos prolongados 2.5.3 Estadia prolongada do paciente
3. Resultados positivos em relação ao processo de enfermagem	3.1 Cuidados de enfermagem hondurenhos éticos e humanizados	3.1.1 Empatia 3.1.2 Apoio emocional 3.1.3 Vocação profissional 3.1.4 Compromisso trabalhista
	3.2 Planejamento de alta com incorporação de autocuidado	

**Figura 2:** - Definições dos grupos temáticos referentes às percepções dos enfermeiros hondurenhos sobre a qualidade do atendimento a pacientes com AVC

GT*	**D** ^†^
1. Resultados negativos em relação à dimensão da estrutura das unidades de atendimento ao paciente	Falha em melhorar ou manter a qualidade da assistência de enfermagem aos pacientes hondurenhos com DCV ^‡^ em relação à estrutura da área onde os pacientes estão hospitalizados.
1.1 Espaços insalubres	Espaços intra-hospitalares sem o cumprimento dos protocolos de desinfecção terminal e simultânea por pessoal treinado.
1.2 Sistema de ventilação inadequado	Unidades de pacientes com sistemas de renovação de ar escassos devido a dispositivos em mau estado ou ruidosos e sem entradas de ar fresco.
1.3 Espaço dimensional limitado	Tamanho inconveniente das unidades em relação ao número de pacientes internados.
2. Desafios relacionados aos processos de assistência de enfermagem	Contestar a validade dos processos de cuidados de enfermagem com alto nível de qualidade para pacientes com DCV ^‡^ no sistema público de saúde de um país de médio-baixo custo.
2.1 Manter a privacidade do paciente durante a realização de intervenções de enfermagem	Dificuldades em manter a segurança e a confidencialidade do paciente durante processos complexos de atendimento devido à superlotação e à falta de suprimentos de proteção.
2.2 Falta de suprimentos e equipamentos	Alguns suprimentos e equipamentos necessários para os processos de tratamento de pacientes com AVC não são suficientes ou não existem, o que significa um risco para a recuperação bem-sucedida do paciente.
2.3 Falta de pessoal de enfermagem especializado	Enfermeiros com treinamento inadequado no atendimento a pacientes com AVC, portanto, experiências de trabalho ruins e possíveis níveis mais altos de estresse relacionado ao trabalho e baixo nível de segurança do paciente.
2.4 Equipe de enfermagem insuficiente em turnos rotativos	Não são contratados funcionários suficientes para as áreas críticas, e a intensidade da carga de trabalho é muito complexa para ser gerenciada com segurança nos diferentes turnos.
2.5 Apoio ambíguo de outros departamentos do hospital	Um estado de incerteza intencional, padrão de comportamento ou situação que pode ser interpretado de forma errônea devido à resistência em cumprir suas responsabilidades nos departamentos de laboratório, imagens e radiação. Consequentemente, isso afeta o diagnóstico do paciente e os planos de tratamento, aumentando o tempo de internação.
3. Resultados positivos em relação ao processo de enfermagem	Experiências agradáveis e úteis para os processos de enfermagem no cuidado de pacientes com DCV ^‡^ .
3.1 Cuidados de enfermagem hondurenhos éticos e humanizados	Considerar a condição de saúde e os sentimentos do paciente e de seus familiares para ajustar seu comportamento e cuidar para demonstrar que o sistema de saúde é razoável e flexível. Portanto, a assistência de enfermagem hondurenha não se apóia nas intenções institucionais, mas em atitudes voltadas para o bem-estar do paciente.
3.2 Planejamento de alta com incorporação de autocuidado	Liberação formal do paciente do hospital quando ele aparenta estar em melhor estado de saúde e apto a receber tratamento ambulatorial em casa, com a integração da família nos planos de cuidados. As atividades para promover o autocuidado e preparar os familiares são realizadas durante todo o período de hospitalização e são de responsabilidade da equipe de enfermagem.

*GT = Grupos Temáticos; ^†^D = Definições; ^‡^DCV = Doença cerebrovascular

### Resultados negativos em relação à dimensão da estrutura das unidades de atendimento ao paciente

Espaços insalubres: Os participantes relatam que os serviços são inadequados, pois não possuem um processo de desinfecção adequado e frequente. Em outras palavras, não cumprem o número de desinfecções simultâneas e terminais exigidas pela complexidade dos departamentos de emergência e de clínica médica. Essa situação ocorre porque o pessoal de limpeza não é designado para permanecer nos departamentos e, da mesma forma, realiza a desinfecção simultânea de segunda a sexta-feira, uma vez por dia. Enquanto isso, nos outros dias, são os familiares dos pacientes que realizam essas atividades. Isso compromete a qualidade das instalações porque os familiares dos pacientes não têm treinamento técnico para realizar as atividades de desinfecção adequadas. Deve-se observar que a frequência das desinfecções terminais na instalação não está documentada.

*Há uma falta de higiene... a equipe de limpeza limpa, mas não de forma completa. Como estamos em uma emergência, assim que chega um paciente com um evento cerebrovascular, outros pacientes renais chegam e mancham as paredes, as camas, e não só isso, mas o sangue também fica no chão.* (P1)

*Isso não está certo. Porque eles não vêm limpar isso diariamente ou de vez em quando, mas quando têm tempo... deveria haver uma equipe específica para a área de emergência e eu não vi nenhuma aqui.* (P3)

*O pessoal de limpeza só trabalha pela manhã, de segunda a sexta-feira... os mesmos membros da família nos ajudam na desinfecção.* (P14)

*O pessoal de limpeza, é de apenas um e eles têm mais áreas para limpar.* (P17)

Sistema de ventilação inadequado: Os serviços não contam com um sistema de ventilação adequado, pois os funcionários relatam que não podem abrir a única janela disponível devido ao risco de acesso a vetores infecciosos fora do hospital. Os sistemas de ventilação, como ventiladores e condicionadores de ar, não estão em boas condições, de modo que os familiares dos pacientes precisam trazer seus próprios suprimentos para melhorar as condições de permanência dos pacientes. Deve-se observar que eles não usam os ventiladores que estão nos serviços porque isso implica um risco de poluição sonora e desarmonização do ambiente relacionado ao ruído produzido quando os ventiladores estão em operação.

*A estrutura é deplorável porque não temos boa ventilação. Os aparelhos de ar-condicionado não funcionam... fica um calor absurdo. Isso afeta o desempenho do pessoal... há janelas, mas elas não abrem porque, como estamos presos a outro lado, insetos podem entrar, ou quando está chovendo, esse mesmo vento traz água.* (P1)

*A enfermaria não é bem condicionada... e isso afeta os pacientes porque alguns deles sufocam, ficam com calor, até mesmo para nós, enfermeiras... a área não é ventilada.* (P7)

*Cada paciente traz seu ventilador... mas não é suficiente, e nosso ventilador também faz muito barulho. Usá-lo é poluição sonora.* (P13)

Espaço dimensional limitado: Os participantes relataram que as dimensões dos cubículos, bem como seu número, são limitadas; isso ocorre porque não são proporcionais ao número de pacientes atendidos pelo serviço. Assim, quando a admissão de pacientes é alta, a equipe de enfermagem é obrigada a colocar vários pacientes no mesmo cubículo, sem respeitar a distância adequada entre eles. Essa situação é alarmante devido ao aumento do risco de infecções nosocomiais.

*As dimensões dos cubículos e o número de unidades disponíveis não são adequadas, ... não só recebemos pacientes com AVC, mas também com suspeita de COVID-19, que é a doença mais frequente hoje em dia, e não temos distanciamento adequado para evitar infecções hospitalares.* (P1)

*São colocados mais pacientes do que o necessário em cada cubículo, e isso se deve à alta admissão de pacientes... Os cubículos são muito pequenos em relação ao número de pacientes admitidos. Não há espaço... às vezes, devido às condições dos cubículos, em vez de ajudar o paciente, ele se complica ainda mais.* (P8)

### Desafios relacionados aos processos de assistência de enfermagem

Manter a privacidade do paciente durante a realização das intervenções de enfermagem: Esse é um dos desafios enfrentados pela equipe de enfermagem no atendimento a pacientes com AVC nos departamentos de emergência e de clínica médica. As condições dos serviços não são favoráveis para a alta demanda de pacientes, de modo que eles são obrigados a colocar vários pacientes no mesmo cubículo em unidades diferentes e muito próximas umas das outras. Assim, os familiares e outros visitantes insistem em permanecer na unidade e a equipe de enfermagem não tem telas para cuidar da privacidade dos pacientes, o que interfere na qualidade dos processos de assistência de enfermagem.

*Em alguns casos, temos que fazer procedimentos em particular para cuidar da integridade do paciente, mas os familiares não querem sair do cubículo... na outra unidade, no mesmo cubículo, há outros pacientes com seus familiares, e temos que dar um jeito de cuidar da privacidade do paciente, embora nem sempre seja possível. (P4)*

*O problema é com os membros da família, apesar de dizermos a eles que essa é uma área restrita, sempre há visitantes e parentes com o paciente. (P5)*

*No momento, não há divisórias e há vários pacientes e familiares no mesmo cubículo. (P9)*

Falta de suprimentos e equipamentos: Os departamentos carecem de equipamentos e suprimentos médico-cirúrgicos para oferecer um atendimento de enfermagem de qualidade aos pacientes com AVC. O hospital fornece algumas necessidades básicas; no entanto, a quantidade não é congruente com o número de pacientes atendidos nos departamentos. Da mesma forma, em relação aos equipamentos médico-cirúrgicos, os departamentos não têm quantidade suficiente, portanto, a equipe de enfermagem é forçada a viajar para outros serviços para solicitar seus equipamentos emprestados. Isso implica um gasto de tempo e energia, sem mencionar que os outros departamentos ficam temporariamente sem o equipamento necessário para o atendimento de pacientes críticos.

*Faltam suprimentos. Muitas vezes, não temos nem mesmo itens básicos como seringas, cateteres intravenosos, soluções intravenosas... A administração do hospital nos dá outra vez, mas não o suficiente... e não temos todo o equipamento médico-cirúrgico de que os pacientes com AVC precisam... temos de pedir a outros departamentos que nos emprestem seus equipamentos. Perdemos tempo com esses deslocamentos.* (P1)

*Deveríamos ter mais equipamentos e suprimentos médico-cirúrgicos para oferecer um melhor atendimento aos pacientes com AVC. Para mim, é um desafio trabalhar nessas condições... faltam alguns suprimentos para oferecer um bom atendimento.* (P3)

*O que fazemos é dar a receita aos membros da família para que eles possam comprar o que precisam.* (P12)

Falta de pessoal de enfermagem especializado: Os departamentos de medicina clínica e emergência não contam com pessoal de enfermagem especializado em cuidados críticos e de emergência, suas competências são limitadas ao conhecimento básico de cuidados e a maioria dos funcionários não tem experiência clínica. Consequentemente, a falta de pessoal especializado faz com que os enfermeiros do departamento dependam de outras disciplinas para tomar decisões relacionadas ao atendimento de pacientes com AVC, perdendo sua autonomia profissional e liderança, o que compromete a qualidade do atendimento na dimensão do processo.

*O médico especialista é responsável por vários procedimentos complexos porque não temos enfermeiros especialistas, e isso é um problema... porque eu, por exemplo, não vou ficar tão atento ao ventilador mecânico porque, embora eu tenha conhecimento básico, sempre deve haver um enfermeiro especialista cuidando de pacientes com AVC, e não temos um no departamento.* (P4)

*A equipe de enfermagem do departamento não é especialista e não tem experiência, portanto, não entende a complexidade de um paciente com AVC. A qualidade do atendimento é comprometida, as tarefas são simplesmente executadas.* (P5)

*Esses pacientes com AVC precisam de cuidados especializados... Temos apenas enfermeiros gerais e nem todos eles são treinados profissionalmente porque são funcionários novos... há pacientes que precisam de cuidados que somente enfermeiros especializados podem realizar. Então ficamos dependentes dos médicos para esses processos. A enfermagem perde autonomia como profissional.* (P7)

Equipe de enfermagem insuficiente em turnos de revezamento: Um desafio notável nos departamentos de emergência e medicina clínica é a falta de pessoal de enfermagem para cada turno. Os participantes relatam que há turnos em que há apenas uma enfermeira geral responsável por 14 pacientes graves e, às vezes, ela precisa dividir suas responsabilidades com alunos e auxiliares de enfermagem porque sozinha não consegue atender a todas as necessidades dos pacientes. No entanto, ainda há coisas pendentes para os próximos turnos que enfrentam o mesmo problema, e a qualidade do atendimento aos pacientes com DCV fica comprometida.

*Os alunos da escola de auxiliares de enfermagem estão colaborando, fazendo alguns turnos conosco.* (P2)

*Há falta de pessoal. Às vezes, há apenas um auxiliar de enfermagem, um estudante de enfermagem e um enfermeiro geral... quando há funcionários em férias ou incapacitados, resta apenas uma pessoa por turno.* (P6)

*Nos turnos da tarde, para fazer a rotação do paciente, é muito difícil. Às vezes estou sozinha como enfermeira, talvez consiga o apoio de um membro da família, mas ainda assim é complicado.* (P13)

*Às vezes, há 13 pacientes para uma enfermeira, e estou falando de um departamento que cuida de pacientes em condições críticas. Portanto, não é possível lidar com tantos pacientes... há muitas coisas pendentes.* (P14)

*Precisamos de mais pessoal, pois não apenas temos pacientes com AVC internados para observação, mas também lidamos com outros pacientes que chegam para outras emergências.* (P19)

Apoio ambíguo de outros departamentos do hospital: Os participantes descrevem dificuldades no processo de diagnóstico e tratamento de pacientes com DCV devido ao apoio ambíguo dos departamentos de apoio. A equipe de imagem não realiza todos os procedimentos de diagnóstico necessários, além de ter horários de atendimento restritos. Da mesma forma, a equipe do laboratório não demonstra disponibilidade para fornecer os resultados dos exames solicitados em tempo hábil. Portanto, a equipe de enfermagem dos departamentos de emergência e de medicina clínica precisa investir tempo e esforço para facilitar os processos dos departamentos de apoio para obter os serviços solicitados.

*Às vezes, leva muito tempo para detectar o tipo de DCV que o paciente tem... Aí a gente fica para trás... o raio X não é feito todo dia, eles têm um horário, mas às vezes eles vêm e dizem que estarão disponíveis até as 18h; às vezes eles vêm e dizem: hoje não vamos fazer raio X nem tomografia.* (P5)

*O departamento de laboratório falha 100%, e dependemos deles... Eles demoram muito para entregar os resultados, apesar de nós os ajudarmos a coletar as amostras [...] o departamento de farmácia só nos dá os medicamentos de uso diário de acordo com a prescrição, mas se tivermos uma emergência, dependemos se o pessoal quer dar ou não. Perdemos muito tempo com isso.* (P7)

### Resultados positivos em relação ao processo de enfermagem

Atendimento de enfermagem hondurenho ético e humanizado: Entre os achados positivos do estudo na dimensão do processo está o tratamento humanizado com ética profissional pela equipe de enfermagem ao paciente com DCV nos serviços de emergência e de medicina clínica. Esse tratamento é baseado na empatia, considerando os sentimentos dos pacientes. Transmitir energia positiva ao paciente, comportando-se de maneira encorajadora, dando apoio emocional e mantendo canais abertos de comunicação tanto com os pacientes quanto com os familiares. Esse tipo de comportamento está presente na equipe de enfermagem com compromisso com a recuperação do paciente e vocação para sua profissão.

*Empatia... Se o paciente estiver consciente e olhar para nós com expressões ruins, ele se sentirá mal. Por isso, sempre tentamos ser alegres e compartilhar energia positiva com o paciente.* (P1)

*Sou uma pessoa que trata os membros da família com gentileza... Tento tratar o paciente da maneira como gostaria que minha família fosse tratada... Gostaria de ser visto da mesma forma que olho para os pacientes.* (P4)

*Os pacientes com AVC são vulneráveis, por isso temos que prestar atenção e dar a eles o máximo de apoio possível, conversar com eles... encorajá-los, ... agir com empatia... dar a eles apoio emocional... eles precisam sentir essa empatia por parte dos enfermeiros.* (P12)

*Eu me sinto bem porque escolhi essa profissão de que gosto. Portanto, cuido dos pacientes com AVC com a melhor atitude.* (P14)

Planejamento da alta com incorporação do autocuidado: O processo de planejamento da alta do paciente do serviço de hospitalização para o retorno seguro e autônomo do paciente à sua casa, com a incorporação da família nas atividades de cuidado. Em Honduras, a enfermagem é responsável pelas atividades educacionais e administrativas relacionadas à alta do paciente.

*O paciente e os membros da família são instruídos sobre os cuidados que os pacientes com AVC precisam... dedicamos nosso tempo para que eles saibam o que fazer quando voltarem para casa.* (P6)

Ao mesclar a pesquisa fenomenológica com a teoria da qualidade do atendimento de Donabedian para pacientes com AVE, obtivemos uma compreensão completa das experiências dos pacientes e dos fatores que afetam a qualidade da assistência médica. Por meio da triangulação, identificamos padrões, temas e inconsistências nos dados, informando sugestões para melhorar a qualidade do atendimento ao paciente com AVE. A comparação e a combinação das descobertas da pesquisa fenomenológica, da estrutura de Donabedian e do modelo de atendimento de enfermagem de Antunez Martinez nos permitiram compreender a qualidade do atendimento ao paciente com AVE, tanto das perspectivas dos participantes quanto do ponto de vista da melhoria da qualidade ^(Figura 3)^.

**Figura 3:** - Estrutura teórica para a qualidade do atendimento de enfermagem a pacientes com AVC

Nota: Elaborado pelos autores com base nos resultados do estudo, no modelo de qualidade de atendimento de Donabedian e no modelo de atendimento de enfermagem de Antunez Martinez

## Discussão

Neste estudo, foram obtidos três temas: Resultados negativos em relação à dimensão da estrutura das unidades de atendimento ao paciente, Desafios relacionados aos processos de atendimento de enfermagem e Resultados positivos em relação ao processo de enfermagem. Este estudo com uma amostra de enfermeiros gerais hondurenhos de enfermarias de emergência e de medicina clínica corrobora os resultados de estudos de países de alta renda que exploram as experiências do pessoal de saúde em relação à qualidade do atendimento a pacientes com AVC.

Os pacientes com AVC exigem atendimento especializado, bem como os equipamentos necessários para um diagnóstico rápido, eficaz e oportuno. No entanto, os departamentos hondurenhos de emergência e de medicina clínica não dispõem de todos os equipamentos necessários para prestar os cuidados exigidos para pacientes com AVC, o que causa uma resposta tardia no diagnóstico desse tipo de paciente, dependendo do tipo (hemorrágico ou isquêmico). Consequentemente, o processo de assistência de enfermagem deve continuar. Essas deficiências afetam diretamente a qualidade da assistência aos pacientes com esse diagnóstico, tanto pela falta de equipamentos quanto pela falta de pessoal de enfermagem competente para cuidar deles.

O achado acima é congruente com um estudo realizado no norte da Inglaterra, onde os resultados destacam a falta de recursos e o ambiente como uma barreira ao atendimento de qualidade e à implementação de diretrizes de intervenção^(21)^. Deve-se observar que a maioria dos estudos sobre a qualidade do atendimento é de países desenvolvidos e, portanto, seu foco está na organização do pessoal e em suas competências específicas, e não na falta de suprimentos e espaços inadequados para internação^(22-23)^. Com exceção de um estudo realizado na Suécia, que destaca as questões ambientais, a pesquisa não aborda as necessidades de cuidados específicos dos pacientes com AVC^(24)^. Recomenda-se continuar explorando essa questão nos sistemas de saúde dos países de renda média-baixa.

Em relação à dimensão do processo, os cuidados de enfermagem que os participantes relataram ser desenvolvidos no serviço foram os de cuidados integrados e competências gerais de enfermagem. Portanto, alguns procedimentos complexos são realizados pela equipe médica, o que implica perda de espaço para o pessoal de enfermagem como profissional autônomo e líder dos processos de cuidado.

Com relação ao atendimento dos pacientes^(25)^ com AVC mencionados, são necessárias fortes habilidades e competências clínicas na interação com o paciente e seus familiares. Portanto, o treinamento contínuo é relevante para o desenvolvimento da competência dos profissionais de saúde em cuidados de enfermagem especializados como parte da qualidade adequada do atendimento^(26-28)^.

Um estudo transversal realizado na Inglaterra, Bélgica, Finlândia, Espanha, Irlanda, Islândia e Suíça aponta que a redução das responsabilidades clínicas complexas da equipe de enfermagem e a sobrecarga da equipe médica podem contribuir para mortes evitáveis, prejudicando a qualidade e a segurança do atendimento hospitalar^(29)^. É necessário aplicar estudos para explorar a dinâmica da organização do trabalho de enfermagem com outros profissionais de saúde para entender esse fenômeno, bem como aplicar estudos quantitativos para medir e descrever as competências dos enfermeiros no sistema de saúde pública latino-americano. Posteriormente, recomenda-se a elaboração de políticas públicas que garantam mais espaços para intervenções avançadas para o pessoal de enfermagem, bem como a promoção de competências especializadas no cuidado crítico de pacientes com AVC.

Da mesma forma, o apoio ambíguo de outros serviços, como os de laboratório e de raio X, é destacado nas narrativas dos participantes. Esse fato é congruente com os resultados de outros estudos que destacam como barreira ao atendimento de qualidade a resposta tardia dos serviços de apoio ao diagnóstico e tratamento do AVC^(30-32)^. Esse problema, assim como o do nosso estudo, deve-se à resistência da equipe de saúde em mudar sua atitude e ao número limitado de funcionários.

O fenômeno acima não é congruente com os princípios do modelo de Donabedian^(11)^, que afirma que a avaliação da qualidade do processo é o conjunto de ações que devem ser realizadas para alcançar um resultado específico. Trata-se de um processo complexo de interação ou comunicação entre o paciente e a equipe de saúde, além da tecnologia utilizada, das competências especializadas exigidas pelo paciente pela equipe de saúde e da execução oportuna dos procedimentos de diagnóstico e tratamento dos pacientes, que devem desempenhar um papel relevante.

Entre os achados positivos, destacam-se a postura ética profissional e o tratamento humanizado dos pacientes com AVE pela equipe de enfermagem. Da mesma forma, os pacientes com AVE precisam de melhor acesso ao apoio emocional por parte da equipe de saúde, incluindo informações e aconselhamento, tanto para os pacientes quanto para os familiares, o que é congruente com as ações dos enfermeiros hondurenhos focadas no tratamento humanizado e no fortalecimento do autocuidado durante o processo de alta^(32-34)^. São necessárias mais pesquisas para estabelecer a eficácia do apoio emocional dos enfermeiros e suas competências em saúde mental.

A importância da educação do paciente e da família durante a alta é reforçada por um autor que menciona que o processo de recuperação após um AVC é complexo e tedioso, portanto, é necessária uma boa orientação sobre as próximas etapas a serem seguidas^(35)^. Essa atividade educacional é priorizada pelos participantes e, posteriormente, recomenda-se explorar essas atividades e o protocolo estabelecido para a alta de pacientes com AVC de acordo com as perspectivas da enfermagem.

As limitações incluíram o fato de que o pessoal de enfermagem que trabalha nos departamentos de emergência e de medicina clínica do HGA não estava acostumado a participar desse tipo de abordagem de pesquisa; consequentemente, isso causou certo grau de estresse porque foi totalmente gravado, limitando, às vezes, a expressão espontânea e completa das respostas às perguntas feitas.

Da mesma forma, o nível de preparação do participante foi uma limitação. Considera-se que o desenvolvimento dessa metodologia com enfermeiros de emergência e de cuidados intensivos pode contribuir para a elaboração de um modelo ampliado para a prática de enfermagem no atendimento a pacientes com AVC. Por fim, o estudo foi realizado em um único local, o que limita uma visão mais geral do fenômeno em Honduras.

Entre os pontos fortes estava o número de participantes, o que permitiu aumentar a representatividade do estudo. Da mesma forma, a pessoa responsável por conduzir as entrevistas foi uma enfermeira com experiência em estudos qualitativos que trabalha na mesma instituição, o que facilitou a aceitação das pessoas contatadas para participar do estudo.

## Conclusão

As experiências dos enfermeiros hondurenhos revelaram um cenário desafiador no setor de saúde para pacientes com AVC. A infraestrutura existente não consegue atender aos padrões básicos de qualidade, colocando em risco a segurança do paciente. Isso lança luz sobre a realidade dos serviços hospitalares em Honduras e as exigentes condições de trabalho enfrentadas pelos enfermeiros, muitas vezes negligenciadas, mas que oferecem percepções aplicáveis à melhoria de situações em todo o mundo. Os enfermeiros dos departamentos de emergência e clínicos de Honduras não têm treinamento especializado em cuidados críticos, o que representa uma limitação que afeta a qualidade do atendimento prestado, dada a complexidade das necessidades dos pacientes com AVC. Há uma necessidade urgente de garantir que os departamentos de emergência contem com pessoas equipadas para liderar os processos de atendimento de forma eficaz. Apesar desses desafios, surge uma nota positiva na dimensão da humanização e do tratamento ético, com a equipe de enfermagem demonstrando apoio e empatia cruciais, fatores que merecem consideração.

Ao triangular a pesquisa fenomenológica com a teoria de Donabedian, é possível obter uma compreensão mais profunda da complexa interação entre as experiências dos enfermeiros e a qualidade dos cuidados de saúde no contexto dos cuidados com o AVC, o que, em última análise, leva a intervenções mais informadas e eficazes para melhorar os resultados e a satisfação dos pacientes. Os formuladores de políticas e gerentes de saúde devem reconhecer a necessidade de incorporar enfermeiros com competências avançadas no processo de atendimento para garantir a recuperação do usuário com o mais alto nível de qualidade de atendimento de enfermagem em países de renda média-baixa. Da mesma forma, ter estruturas organizacionais claras e melhor gerenciamento de recursos. Esses tipos de estratégias trarão maiores benefícios para as instituições de saúde pública e aumentarão a satisfação do usuário, assim como reduzir a permanência hospitalar.
